# The bread wheat epigenomic map reveals distinct chromatin architectural and evolutionary features of functional genetic elements

**DOI:** 10.1186/s13059-019-1746-8

**Published:** 2019-07-15

**Authors:** Zijuan Li, Meiyue Wang, Kande Lin, Yilin Xie, Jingyu Guo, Luhuan Ye, Yili Zhuang, Wan Teng, Xiaojuan Ran, Yiping Tong, Yongbiao Xue, Wenli Zhang, Yijing Zhang

**Affiliations:** 1 National Key Laboratory of Plant Molecular Genetics, CAS Center for Excellence in Molecular Plant Sciences, Shanghai Institute of Plant Physiology and Ecology, Shanghai Institutes for Biological Sciences, Chinese Academy of Sciences, 300 Fenglin Road, Shanghai, 200032 China; 2 University of the Chinese Academy of Sciences, Beijing, 100049 China; 3 State Key Laboratory for Crop Genetics and Germplasm Enhancement, Jiangsu Collaborative Innovation Center for Modern Crop Production, Nanjing Agricultural University, No.1 Weigang, Nanjing, 210095 Jiangsu China; 4 Henan University, School of Life Science, Kaifeng, 457000 Henan China; 5 The State Key Laboratory of Plant Cell and Chromosome Engineering, Institute of Genetics and Developmental Biology, the Innovative Academy of Seed Design, Chinese Academy of Sciences, Beijing, 100101 China

**Keywords:** Bread wheat, Allohexaploid, Epigenomic map, Chromatin signature, Regulatory element, Promoter, Enhancer

## Abstract

**Background:**

Bread wheat is an allohexaploid species with a 16-Gb genome that has large intergenic regions, which presents a big challenge for pinpointing regulatory elements and further revealing the transcriptional regulatory mechanisms. Chromatin profiling to characterize the combinatorial patterns of chromatin signatures is a powerful means to detect functional elements and clarify regulatory activities in human studies.

**Results:**

In the present study, through comprehensive analyses of the open chromatin, DNA methylome, seven major chromatin marks, and transcriptomic data generated for seedlings of allohexaploid wheat, we detected distinct chromatin architectural features surrounding various functional elements, including genes, promoters, enhancer-like elements, and transposons. Thousands of new genic regions and cis-regulatory elements are identified based on the combinatorial pattern of chromatin features. Roughly 1.5% of the genome encodes a subset of active regulatory elements, including promoters and enhancer-like elements, which are characterized by a high degree of chromatin openness and histone acetylation, an abundance of CpG islands, and low DNA methylation levels. A comparison across sub-genomes reveals that evolutionary selection on gene regulation is targeted at the sequence and chromatin feature levels. The divergent enrichment of cis-elements between enhancer-like sequences and promoters implies these functional elements are targeted by different transcription factors.

**Conclusions:**

We herein present a systematic epigenomic map for the annotation of cis-regulatory elements in the bread wheat genome, which provides new insights into the connections between chromatin modifications and cis-regulatory activities in allohexaploid wheat.

**Electronic supplementary material:**

The online version of this article (10.1186/s13059-019-1746-8) contains supplementary material, which is available to authorized users.

## Background

The completion of a high-quality bread wheat reference genome sequence [1] provided researchers with a good opportunity for a thorough analysis of gene regulation in hexaploid wheat. A previous study indicated that the noncoding regions account for approximately 93% of the 16-Gb genome [1]. Studies in both higher plants and animals revealed abundant highly conserved regulatory elements in noncoding regions with essential regulatory activities [2, 3], the detection of which is critical for a comprehensive characterization of the regulatory networks. Conserved sequences are a typical feature of regulatory elements. However, considering the intrinsic shortness and degeneracy of sequence motifs underlying regulatory activities, the detection of functional elements merely based on sequence information for large intergenic regions is far from accurate. It is well-acknowledged that the interplay between cis-regulatory elements and epigenetic modifications is crucial for regulating gene activity. In humans, the major regulatory elements, including promoters, enhancers, and transcription factor-binding sites, are largely predictable based on chromatin features [4]. Various types of chromatin modifications regulating gene activity have been described and applied for pinpointing cis-regulatory elements, including histone acetylation; mono-, di-, and tri-methylations; and DNA methylation [5]. Additionally, chromatin openness has been characterized with various techniques to identify regions that are highly accessible to regulatory factors [6]. The “epigenetic code hypothesis” that has been proposed states that different combinations of chromatin modifications are associated with distinct biological consequences [7]. Thus, the integration of epigenomic architecture and sequence features has become a powerful means for pinpointing the regulatory elements in the genome and for revealing the regulatory mechanism, ultimately helping to accurately narrow down the chromosomal locations of candidate functional regions [4, 5, 8–11].

Because of its recent history, hexaploid wheat is a good model plant species for studying polyploidy, the major factor driving the evolution of plant genomes [12]. Alterations in the epigenetic and transcriptional regulation of duplicated gene copies are among the main factors contributing to the increased developmental flexibility and adaptability of bread wheat to diverse environments [13–15]. In a recent study, the transcriptome of bread wheat was comprehensively profiled across a range of tissues and cultivars, which systematically characterized the similarities and differences in the expression of homologs [13]. Epigenetic modification is one major component regulating transcription, and how epigenetic modifications, especially those localized in intergenic regulatory regions, influence the transcription across sub-genomes remains unclear. Furthermore, many gene families expanded during hexaploidization [1], and the epigenetic regulation of the newly generated gene copies and the ancient genes is an interesting issue that may be correlated with the advantages of polyploidy. To address these issues, a systematic characterization and comparison of epigenomic architectural features across hexaploid wheat sub-genomes is indispensable.

In the present study, the epigenomic maps of open chromatin, multiple histone modifications, and DNA methylation, as well as transcriptomic patterns were systematically profiled. We detected the distinct chromatin architectural and sequence features associated with different functional elements. Further comparisons across sub-genomes revealed highly conserved sequence and epigenomic architecture surrounding a subset of active regulatory regions. Additionally, the extent of the chromatin openness and H3K9 acetylation marks was highly correlated with enhancer activities, as reflected by a reporter assay, demonstrating that the specific combinatorial epigenomic pattern is a good predictor of cis-regulatory elements in bread wheat.

## Results

### Chromatin architecture of bread wheat seedlings

**Fig. 1:**
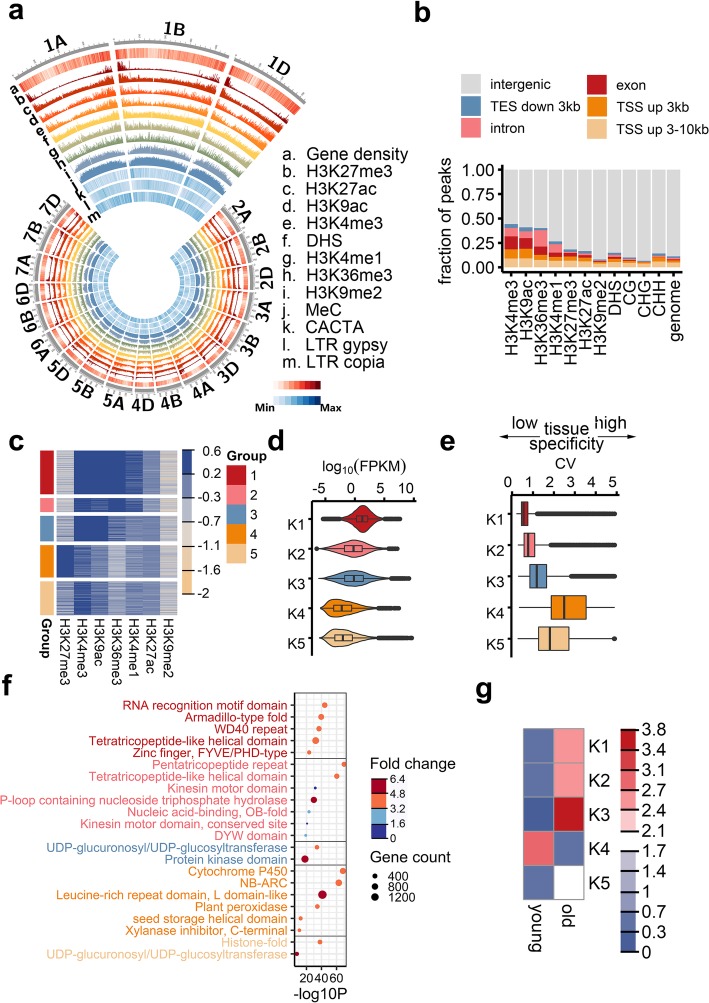
Chromatin profiling revealed the epigenetic regulation of genes. **a** Circos plot summarizing the chromosomal distribution of epigenetic marks. The outermost circle depicts the ideograms of each chromosome. The second outermost circle represents gene density, with red and white indicating high and low density, respectively. Bar plots in the middle circles present the density of epigenetic marks, including seven histone modifications, DNase I-hypersensitive site (DHS), and DNA methylation levels. The three innermost circles represent the densities of three major transposable elements (TEs) in wheat (CACTA, Gypsy, and Copia). **b** Peak distribution of each mark surrounding various genomic features. TSS, transcription start site; TES, transcription end site. **c** Five groups of genes marked by different combinations of epigenetic modifications. The normalized intensity of each mark surrounding genes was recorded for *k*-means clustering. **d** Violin plot presenting the distribution of gene expression densities for various groups. **e** Boxplot presenting the tissue specificity, which is represented by the coefficient of expression variance (CV) across various tissues. The transcriptomic data for seven tissues were published previously [13]. **f** Top enriched protein families for each gene group ranked based on the enrichment *P* value. Each circle represents one enriched term. The color intensity of the circle represents the fold enrichment. The size of the circle represents the number of genes in each group with the given term. **g** Enrichment of each gene group for conserved genes (old) or non-conserved genes (young)

To systematically analyze the epigenomic features in bread wheat, we profiled the open chromatin associated with DNase I-hypersensitive sites (DHSs), the DNA methylome at a single-base resolution based on bisulfite sequencing, and the genomic distribution of seven histone modifications according to chromatin immunoprecipitation followed by high-throughput sequencing (ChIP-sequencing) in seedlings (see the “Methods” section). We used specific antibodies to detect histone H3 lysine 4 tri-methylation (H3K4me3), a modification that generally occurs in promoters; H3K27me3 associated with repression; lysine 9 acetylation (H3K9ac) and H3K27ac associated with active regulation, mostly in promoters and enhancers; H3K36me3 associated with transcribed regions; H3K9me2 responsible for the repression of transposable elements (TEs); and H3K4me1, which is preferentially associated with enhancers in vertebrates, but in gene body regions in *Arabidopsis thaliana* [16] (Fig. 1a and Additional file 1: Figure S1). All data were visualized with a customized genome browser (http://bioinfo.sibs.ac.cn/cs_epigenome). Among these marks, H3K9me2 and DNA methylation are primarily localized to centromere-proximal regions, while other marks, which are mainly involved in regulating gene activities, are mostly localized to interstitial and distal end regions with high gene densities (Fig. 1a). For these gene regulatory marks, more than half of the peaks corresponded to intergenic regions (Fig. 1b), which is a proportion that is much higher than that of previously well-characterized model plant species with relatively small genomes, including *A. thaliana* and rice. These results suggested that these marks are potentially involved in the remote regulation of gene activity.

### Chromatin signatures surrounding genes

We started by characterizing the chromatin features surrounding promoter and genic regions, as well as their association with gene activities. The genes targeted by these marks were divided into five groups based on the combination of marks (Fig. 1c and Additional file 2: Table S1). Highly expressed genes (group I) were preferentially marked by H3K4me3, H3K9ac, and H3K36me3 (Fig. 1d), similar to the pattern reported in vertebrates, bread wheat, and other plant species [13, 17, 18]. The expression of group 4 genes preferentially marked by H3K27me3 exhibited high tissue specificity (Fig. 1e), consistent with the role of the Polycomb group proteins (PcGs) responsible for catalyzing H3K27 tri-methylation to control development [19]. The most enriched protein families in each group are presented in Fig. 1f. Interestingly, nucleotide-binding site leucine-rich repeat (NB-LRR) genes accounted for 30% of the group 4 genes, which were marked by H3K27me3, implying that PcGs may influence wheat immunity.

The polyploidization of wheat resulted in the expansion of many gene families [1], which generated an abundance of new genes. We wondered how these newly generated gene copies are regulated at the epigenetic level. We divided the bread wheat genes into the following two categories: “old” genes within regions highly similar to the genomes of diploid and tetraploid progenitors, and “young” non-conserved genes localized to regions with little or no similarities to the genomes of wild progenitors (see the “Methods” section). We observed a substantial enrichment of “young” genes in groups with H3K27me3 marks (Fig. 1g). Because H3K27me3 preferentially occurs at the distal end of chromosomes with a high recombination rate [1] (Fig. 1a), it is likely that H3K27me3 repressed new genes in specific genomic regions.

### Association between sub-genome divergence in gene expression and promoter histone marker density

**Fig. 2:**
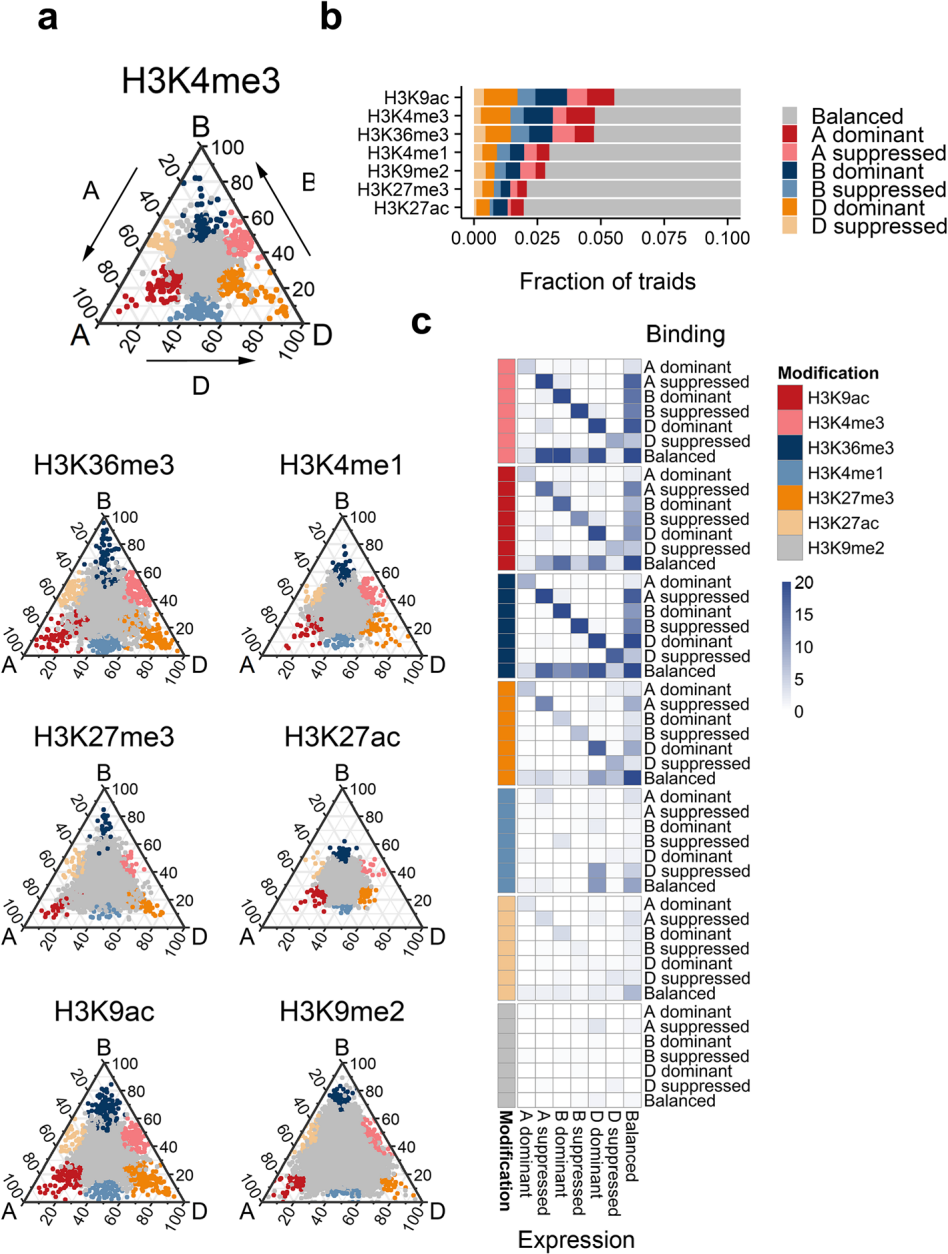
Sub-genome-biased promoter binding and regulation of homolog triads by various markers. **a** Ternary plot presenting the relative binding densities of seven epigenetic marks in the promoters of triad genes. Each circle represents a gene triad. The distance for each triad was determined based on the ratio of the normalized read density for one sub-genome to the read density for all sub-genomes. **b** Fraction of triads with significantly unbalanced binding across sub-genomes. **c** Enrichment of the overlap between the biased binding of epigenetic marks and the biased expression of target genes. Dark blue represents a significant overlap. A total of 12,669 expressed triad genes were used

Bread wheat is an allohexaploid species with three sub-genomes. The divergence of redundant gene copies would be expected to increase the adaptability of bread wheat [20]. To examine the sub-genome preferential binding of the epigenetic marks in promoter regions and the associated functional consequences, a previously described ternary plot was applied [13], with a particular focus on 12,669 expressed triads (1.1:1 correspondence across the three homologous sub-genomes). All triads were divided into the following seven sub-genome-biased binding groups: a balanced group, with similar binding across the three homologs, and six dominant or suppressed groups, with higher or lower binding in one homolog (Fig. 2a and Additional file 2: Table S2). Among these modifications, the binding of H3K4me3, H3K9ac, and H3K36me3 was highly diverse, whereas the other modifications were relatively stable across the three sub-genomes (Fig. 2b). The sub-genome-biased binding was further compared with sub-genome-biased expression. We observed that the divergent binding of H3K4me3, H3K9ac, and H3K36me3 was highly correlated with the biased expression of the target genes (Fig. 2c). A recent study revealed the positive correlation between sub-genome divergence in transcription and H3K36me3/H3K9ac binding across gene body regions. In the current study, we determined that these promoter region modifications were also closely associated with sub-genome divergent transcription. Moreover, we identified the top active histone modifications whose divergent binding is closely associated with divergent transcription among the major regulatory histone marks.

### Distinct and predictive chromatin features surrounding various functional elements

**Fig. 3:**
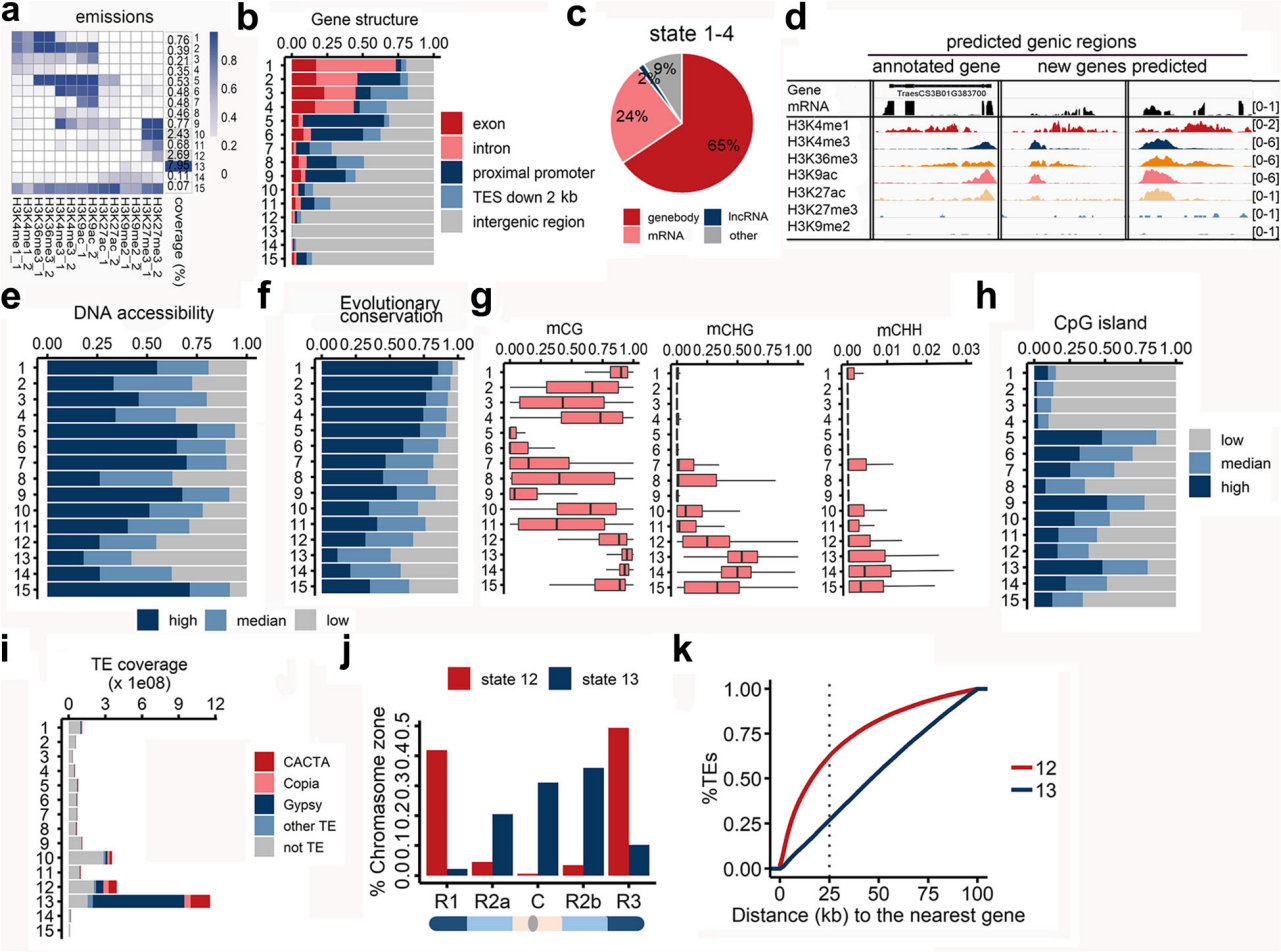
Distinctive and predictive chromatin signatures of functional elements. **a** Chromatin states determined with a multivariate hidden Markov model. The heatmap presents the emission parameters based on genome-wide combinations of epigenetic marks. Dark blue represents a high frequency of a given mark at regions corresponding to the chromatin state. Each row represents one state, and each column represents one chromatin mark, except the last column, which represents the genomic coverage of the given state. Replicates displayed good consistency. **b** Bar plot indicating the distribution of genomic positions in each state for various genomic features. **c** Pie plot presenting the fraction of regions in each chromatin state covering mRNA or lncRNA sequencing reads. **d** Genomic tracks illustrating three predicted genes based on the signatures of chromatin states 1–4. The first gene was annotated according to the IWGSC RefSeq genome assembly (version 1.0), whereas the other two genes were not annotated, but had a high RNA-seq read density. The data in square brackets represent the range of normalized read densities. The number of reads in each position was normalized against the total number of reads (reads per million mapped reads). **e**, **f** For each chromatin state, the fractions of open chromatin regions characterized by DHS read density (**e**) and conservation score across wheat species (**f**) were calculated. **g** Boxplot presenting the distribution of DNA methylation ratios of each chromatin state in three sequence contexts. **h** Fraction of each chromatin state overlapping with a CpG island. **i** Number of regions in each chromatin state associated with various types of TEs. **j** Distribution of TEs in various genomic segments associated with distal (R1 and R3) as well as interstitial and proximal (R2 and C) regions in chromatin states 12 and 13. **k** Cumulative distances of TEs to the nearest genes in chromatin states 12 and 13

Bread wheat has extremely large intergenic regions that are five times larger than those of the human genome. These regions are likely rich in distal gene regulatory elements marked by various chromatin architectural features. To summarize the genome-wide combinatorial patterns of these chromatin marks, we applied a multivariate hidden Markov model (HMM) to distinguish between chromatin states [21]. This method has been widely used in human studies, in which it has enabled the detection of regulatory elements related to individual marks [21]. The genomic regions marked by these modifications were divided into 15 chromatin states based on the combinations of marks (Fig. 3a), which were associated with a distinct enrichment of biological activities. All states could be visualized with a customized genome browser (http://bioinfo.sibs.ac.cn/cs_epigenome). To functionally characterize the various chromatin states, the genomic, transcriptomic, and epigenomic features of these 15 states were examined in detail. Specifically, we analyzed the distribution of gene structures, chromatin accessibility, sequence conservation across ploidy levels, coverage of CpG islands (CGIs), and DNA methylation levels (Fig. 3). High read densities for all modifications were observed for chromatin state 15. Subsequent examinations revealed that this state was associated with relatively low conservation regarding sequences and chromatin features (see below). Thus, it is likely that the signal for this state was mostly derived from background noise, and the state was not analyzed further.

Regions corresponding to chromatin states 1–4 were enriched in gene body regions, accounting for 1.7% of the whole genome. These states were mainly characterized by the enrichment of H3K4me1 and H3K36me3, the typical histone marks present in actively expressed genes (Fig. 3a and b). We observed that most (91%) of these regions were transcriptionally active. In addition to 65% of the regions carrying annotated genes, 24% and 2% of the unannotated regions were associated with mRNA and lncRNA sequencing reads, respectively (Fig. 3c). We identified 8987 transcribed regions in states 1–4 that were not annotated in the high-confidence gene models (the coordinates are listed in Additional file 2: Table S3). Further examination revealed that 63% (5660/8987) of the 8987 transcripts belonged to low-confidence gene models, and the rest transcripts mostly (94%) had low coding potential, i.e., non-coding transcripts (Additional file 2: Table S3). Genomic tracks in Fig. 3d illustrates the transcriptional activity (reflected by the RNA-seq read density) associated with the regions in these states, including annotated in the high-confidence gene models (the first panel) and unannotated (the other two panels) regions. Thus, the combinatorial pattern of these active histone marks is a good predictor of actively expressed genes.

Increased nuclease sensitivity and H3K9ac or H3K27ac are typical characteristics of regulatory elements, including promoters and enhancers, as previously reported in humans and maize [22, 23]. Chromatin states 5–7 were typically enriched for H3K9ac, as well as H3K27ac to a lesser extent (Fig. 3a). Some regions in these states also had a high density of H3K4me3 and H3K36me3. The last column in Fig. 3a represents the genome coverage of each state, and states 5–7 with potential regulatory activities accounted for roughly 1.5% of the whole genome [0.53% (state 5), 0.48% (state 6), and 0.48% (state 7)]. These states exhibited extensive chromatin openness characterized by abundant DHS reads (Fig. 3e), thus representing DNA regions that are easily accessed by transcription factors and other regulatory proteins. In addition to a high density of DHSs and histone acetylation, chromatin states 5–7 included several other typical features of transcriptional regulatory elements as revealed in human studies [4, 5, 8, 9]. First, because regulatory elements are crucial for transcriptional regulation, they are generally subjected to purifying selection. A conservation score was calculated for each position of the wheat genome sequence by comparing homologous genomes with various ploidy levels (see the “Methods” section). The conservation scores for the sequences of chromatin states 5–7 were relatively high, similar to those of the gene body regions (Fig. 3f). Second, the DNA methylation levels in chromatin states 5–7 were low, especially in the CG context (Fig. 3g and Additional file 1: Figure S2). Cis-regulatory regions are generally devoid of DNA methylation, which is supposed to be responsible for chromatin compaction and gene silencing [24]. Third, CGIs in mammalian genomes are important genomic elements related to transcriptional regulation and are associated with promoters and enhancers devoid of DNA methylation [25]. Whether the CGI-like regions in plants are functionally equivalent to mammalian CGIs remains controversial [26]. We observed that in wheat, CGIs were generally sheltered from DNA methylation in the CG and CHG contexts, which represent the major types of DNA methylation (Additional file 1: Figure S3). The CGIs were relatively abundant in chromatin states 5–7, especially in state 5 with increased chromatin accessibility and sequence conservation (Fig. 3h). Altogether, these results provide additional evidence that chromatin states 5–7, accounting for approximately 1.5% of the whole genome, include a subset of active regulatory elements.

Our data revealed that chromatin state 13 was the largest and was characterized by the binding of H3K9me2 (Fig. 3a). A reinforcement loop exists between H3K9me2 and DNA methylation to repress TE activities and mediate chromosome condensation [27]. The regions in chromatin state 13 consistently had high DNA methylation levels (Fig. 3g), high TE abundance (Fig. 3i), and low DHS densities (Fig. 3e). Interestingly, instead of an enrichment of the H3K9me2 mark, a subset of TEs was preferentially marked with H3K27me3 (chromatin state 12). A subsequent examination revealed that considerably fewer Gypsy-type LTR sequences were detected for H3K27me3-marked TEs (chromatin state 12) than for TEs marked by H3K9me2 (chromatin state 13) (Fig. 3i). Because Gypsy sequences are mostly localized to centromere-proximal regions, we examined the chromosomal localization of TEs preferentially marked by H3K27me3 and H3K9me2. As expected, TEs marked by H3K27me3 were preferentially localized to the distal end of chromosomes with high gene densities, whereas TEs repressed by H3K9me2 were localized to centromere-proximal regions (Fig. 3j, k). It is well-acknowledged that H3K9me2 and DNA methylation are responsible for the stable repression of TEs, whereas H3K27me3 is related to a conditional or relatively transient repression responsible for controlling developmental transitions or environmental stress responses. Thus, it is likely that the repression of TEs in gene-abundant regions by H3K27me3 may restrict the condensation of chromosomal regions surrounding genes.

### Conservation of chromatin architecture surrounding functional elements across sub-genomes

**Fig. 4:**
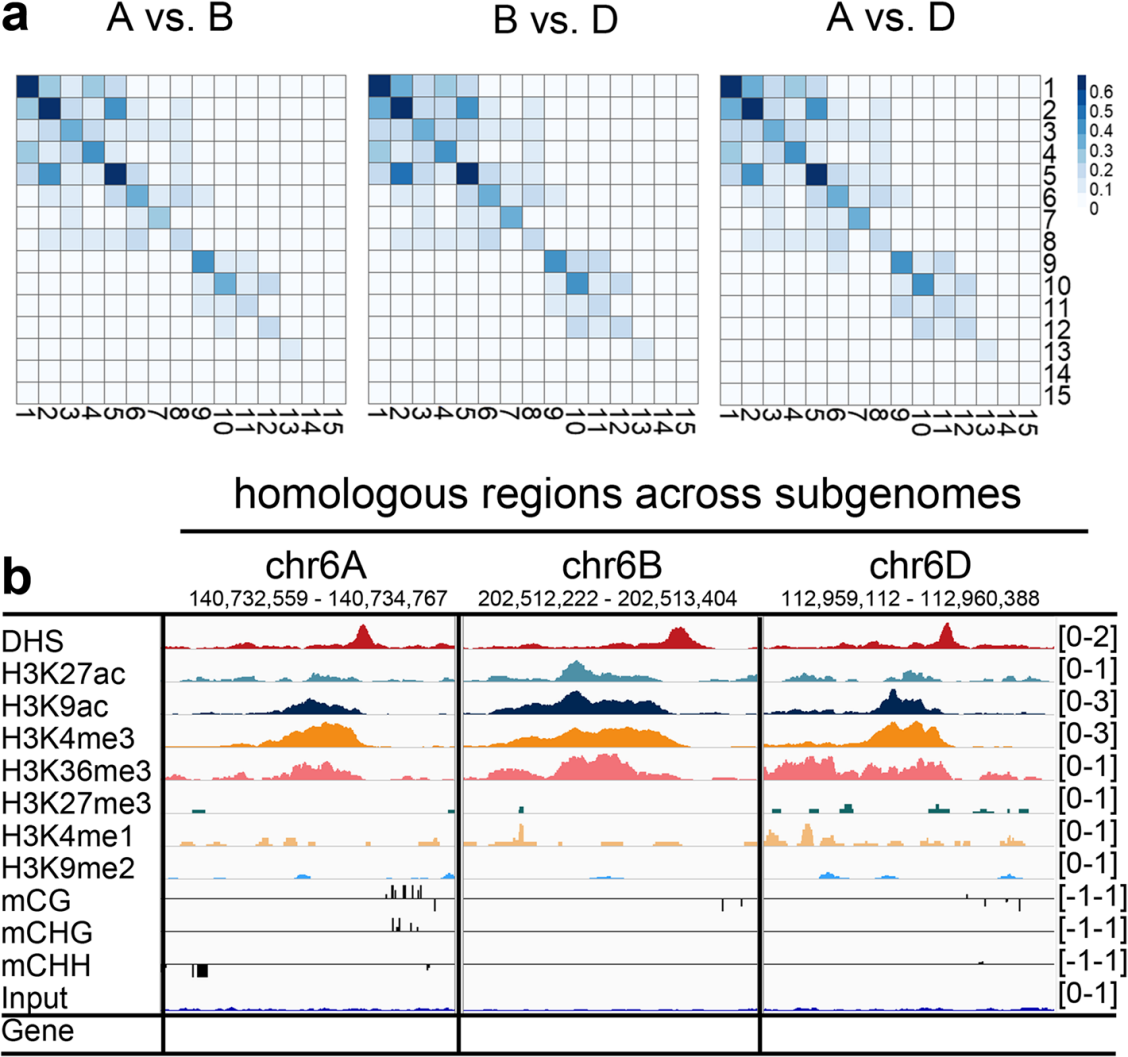
Conservation of the epigenetic architecture of regulatory elements across sub-genomes. **a** Pair-wise comparison (Jaccard similarity) of shared chromatin states between sub-genomes (i.e., the fraction of sub-genome collinear regions from each chromatin state sharing the same state between sub-genomes). **b**. Genomic tracks illustrating the conservation of epigenetic features in the predicted enhancer regions exhibiting sequence collinearity across three sub-genomes. The number of reads in each position was normalized against the total number of reads (reads per million mapped reads)

To further estimate the conservation and diversity of chromatin signatures across sub-genomes at the epigenomic level, we assessed the similarities of distinct chromatin states between sub-genomes, with a focus on collinear regions. The chromatin states were largely similar across the three sub-genomes, with the exception of chromatin states 14 and 15 (Fig. 4a). Chromatin state 5 corresponding to regulatory elements and chromatin states 1 and 2 corresponding to gene body regions were highly similar across the three sub-genomes, with Jaccard similarity indices ranging from 0.64 to 0.67. Genomic tracks in Fig. 4b illustrates one intergenic collinear region of the three sub-genomes with a similar epigenetic pattern. Regions in these states are most likely functionally conserved given the highly conserved sequence and epigenetic activity.

### Common and unique features of promoters and enhancer-like elements

**Fig. 5:**
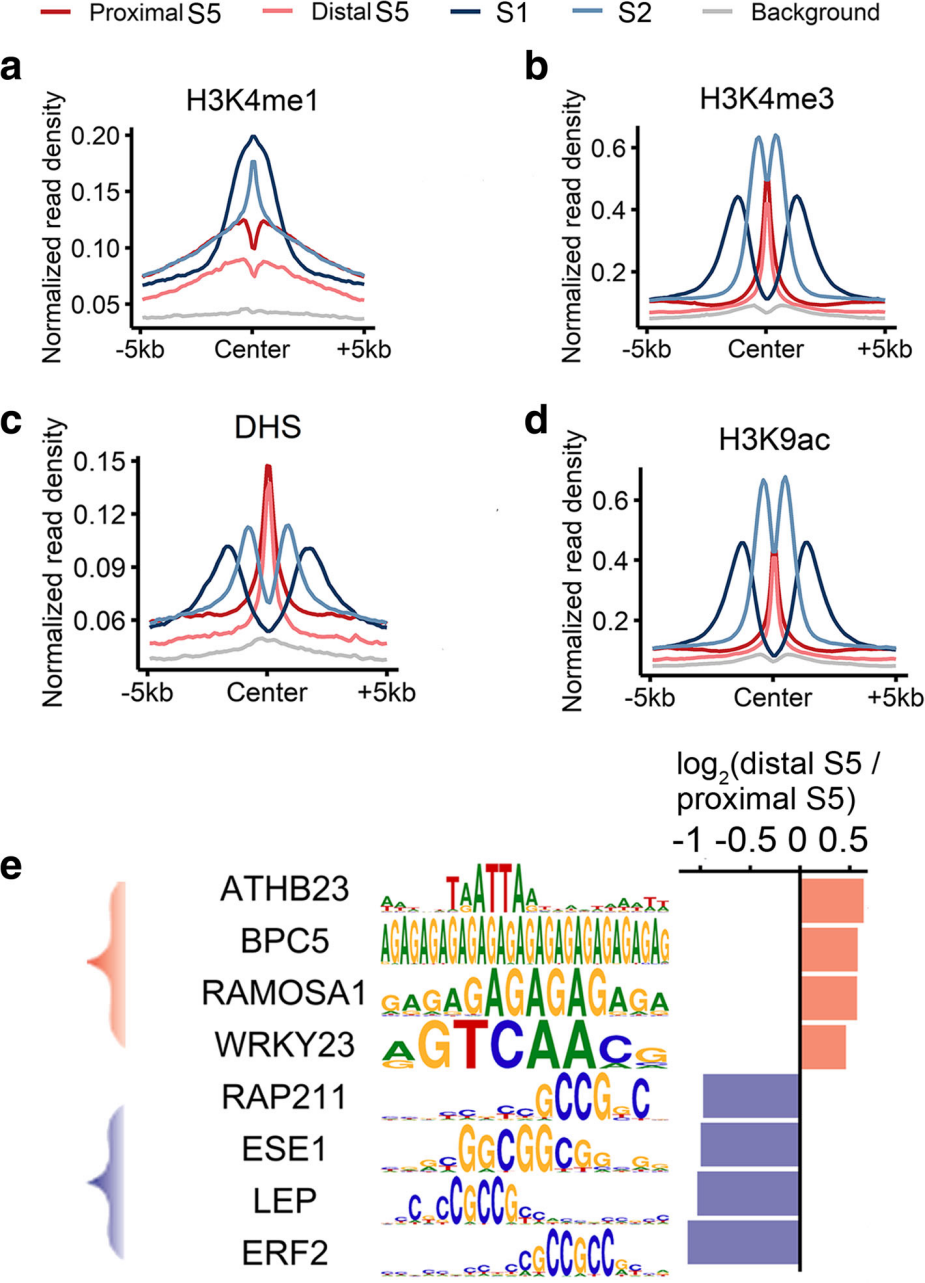
Epigenetic and sequence features of predicted promoters and enhancers. **a–d** Average profile of H3K4me1 (**a**), H3K4me3 (**b**), DHS (**c**), and H3K9ac (**d**) read densities surrounding the center of gene-proximal and gene-distal regions in chromatin state 5 (S5) and regions in chromatin states 1 (S1) and 2 (S2) corresponding to gene bodies highly conserved across three sub-genomes. **e** Cis-elements differentially enriched in the promoter and enhancer-like regions in state 5. The bar plot represents the relative log_2_ fold-change of the occurrence of corresponding motifs in promoters versus enhancer-like regions

To examine the differences between proximal promoter and distal regulatory elements, we compared the sequence features and chromatin signatures between these two types of regions in chromatin state 5, which displayed typical epigenetic features of cis-regulatory elements and are highly conserved across subgenomes (Figs. 3a, 5a–d, and Additional file 2: Table S4). On average, H3K4me1 was more extensive surrounding promoters than in enhancer-like elements in wheat seedlings (chromatin states 1 and 2 in Fig. 5a). This is in contrast to the findings in humans, in which H3K4me1 is reportedly significantly enriched in enhancer regions [4], and enhancers are typically characterized by high density ratio  of H3K4me1 to H3K4me3 [4]. However, in wheat, H3K4me3 was more abundant than H3K4me1 in enhancer-like regions (chromatin state 5 in Fig. 5a, b). Therefore, the regulatory roles of H3K4 modifications appear to have evolved independently between plants and animals. Both proximal and distal elements were associated with similar levels of chromatin openness and H3K9ac densities (Fig. 5c, d). To further discriminate between promoter and enhancer-like sequences, we performed a cis-element enrichment analysis for the proximal (promoter) and distal (enhancer-like) regulatory elements in state 5 (Fig. 5e and coordinates listed in Additional file 2: Table S4). Different sets of transcription factor-binding motifs were over-represented in the promoters and enhancer-like elements. The promoters were enriched with GCC/GGC-rich motifs, whereas the enhancer-like elements tended to have GA/TC- and AT/TA-rich motifs. Similar results were obtained in earlier human studies, which concluded that the GA dinucleotide repeat is required for and may be used to predict broadly active enhancers [28, 29]. This differential preference for sequence contexts between promoter and enhancer-like regions may reflect the diversity in the transcription factor occupancy between these two types of regulatory elements.

### Functional validation of enhancer-like elements

**Fig. 6:**
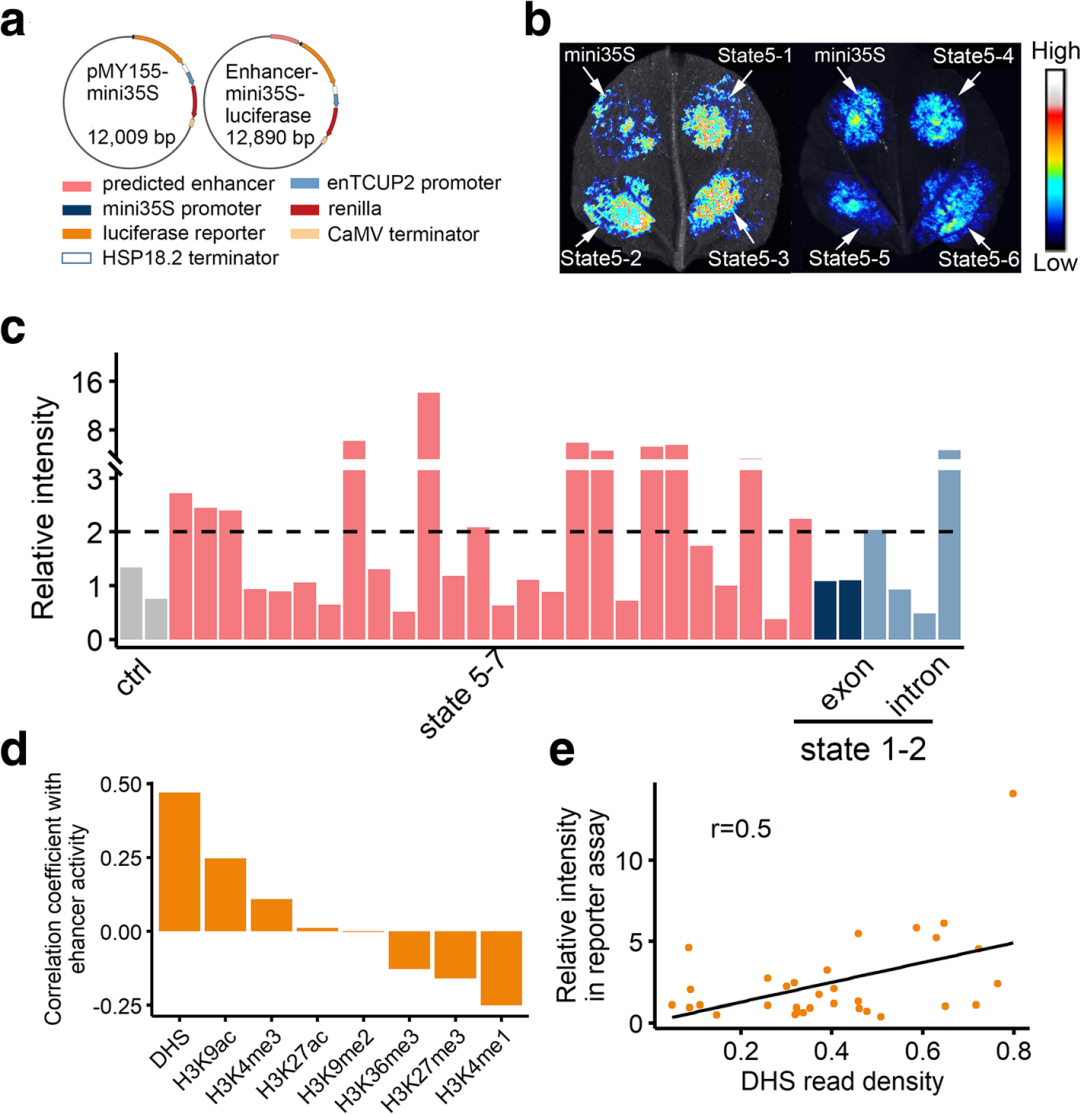
Experimental validation of predicted enhancers. **a**, **b** The predicted enhancer loci were cloned into pMY155 reporter constructs (**a**) to examine their regulatory potential based on a luciferase reporter assay (**b**). Results for the first six predicted regions are presented, and results for the other 26 predicted regions are provided in Additional file 1: Figure S4. **c** Relative intensity of 26 predicted enhancer-like elements in states 5–7 and six active genic regions in states 1 and 2 in the reporter assay. Two control regions (ctrl) were randomly chosen from genomic regions without any histone modifications characterized in the present study. The order of the region tested is the same with the experimental results shown in Additional file 1: Figure S4 and the quantitative results listed in Additional file 2: Table S5. **d** Correlation between the relative intensity determined by the reporter assay and the ChIP-sequencing or DNase-sequencing read density in predicted enhancer-like regions of each epigenetic mark. Detailed data are listed in Additional file 2: Table S5. **e** Scatter plot presenting the correlation between the DHS read density and relative intensity in the reporter assay for the 32 predicted regions. Detailed data are listed in Additional file 2: Table S5

We further verified the functional potential of the predicted enhancer-like elements based on a luciferase reporter assay. Twenty-six gene-distant sequences (distance to the nearest gene > 20 kb) in chromatin state 5 with varying DHS densities were analyzed. Specifically, these sequences were inserted into a reporter vector under the control of the 35S promoter, after which the recombinant plasmids were used for the transient transfection of *Nicotiana benthamiana* leaves (Fig. 6a). Of the 26 evaluated sequences, half of the regions exhibited robust activity in the reporter assay, with higher signals in all three spots in all three replicates compared with the controls carrying the 35S promoter alone. Figure 6 b and c and Additional file 1: Figure S4 present the results of one replicate. The data for all replicates are listed in Additional file 2: Table S5. In mammals, some intronic regions may enhance the expression of nearby genes [30, 31]. To test if wheat intronic regions with an active epigenetic architecture can enhance gene expression, we examined the enhancer activity of regions in states 1 and 2 with high DHS density, including four intronic and two exonic regions. Among these six regions, two of the intronic regions displayed robust activity (Fig. 6c, Additional file 1: Figure S4, and Additional file 2: Table S5), indicating the wheat introns exhibit enhancer activity. To further determine which epigenetic marks are correlated with the enhancer activity, we calculated the Pearson correlation coefficients for the signal densities from the reporter assay and the read densities of each epigenetic mark in the tested regions. Enhancer activity was most correlated with DHS and H3K9ac densities (*r* = 0.5 and 0.3, respectively) (Fig. 6d, e), indicating these marks are good predictors of enhancer activity.

## Discussion

In this study, we used combinatorial patterns of chromatin features to analyze functional elements in hexaploid wheat, especially the regulatory elements in large non-coding genomic regions. We revealed that approximately 1.5% of the genome potentially encodes a subset of active cis-elements, including promoters and enhancer-like elements. Among these regions, state 5 was highly conserved regarding the sequence and epigenetic activity across the sub-genomes, indicating that the purifying selection on gene regulation involves both sequence and chromatin features. These findings significantly narrowed down the possible locations of functionally pivotal regions within the large intergenic regions of the bread wheat genome.

Comparing the conservation and divergence of transcriptional regulation between different ploidy levels is critical for a thorough understanding of the functional consequences of polyploidization, the major driving force for plant evolution [12]. Because of a recent hexaploidization event in bread wheat, the divergence between hexaploid wheat and its progenitors is not very large, thus enabling an effective comparison across wheat of different ploidy levels. To characterize the gene regulatory mechanisms, a systematic and accurate annotation of regulatory elements is a prerequisite. However, the large intergenic regions of the bread wheat genome represent a major challenge for researchers attempting to pinpoint functionally important elements and reveal regulatory mechanisms. Previously well-studied model plant species, including *A. thaliana* and rice, have genomes with only 100–400 million base pairs, and the available information regarding distal transcriptional regulation in plants is limited. The relationship between chromatin features and transcriptional regulatory activities revealed in the present study may be useful for predicting cis-regulatory elements and regulatory activity in wheat genomes.

The chromatin architectural features surrounding functional elements in the wheat genome share similarities and differences with those in mammalian genomes. First, CGIs have been well-characterized in mammals as important regulatory regions devoid of methylated DNA [26], whereas the functions of plant CGI-like regions remain unclear [26]. We observed that similar to the epigenetic patterns of CGIs in mammals, the CGIs in wheat were generally lacking in the major form of DNA methylations (Additional file 1: Figure S5) and many of them were present in active cis-regulatory regions (Fig. 3h). In addition to states 5–7, CGIs were abundant in state 9, which was associated with a high density of H3K27me3. This is consistent with a previous report that indicated CGIs in animals can be repressed by a PcG-mediated H3K27me3 modification [26]. However, we also detected a subset of CGIs enriched in state 13 preferentially marked by H3K9me2, which contradicts the results of an earlier animal study revealing the depletion of H3K9me2/3 surrounding CGI+ genes [32]. This may be associated with the difference of the epigenetic factors involved in catalyzing H3K9 di-/tri-methylation between animals and plants [33]. Second, similar cis-elements were over-represented in enhancer-like elements in wheat and mammals, with a GA dinucleotide repeat as a typical example (Fig. 5e), indicating a likely ancient regulatory mechanism for distal gene regulation. Third, similar to the pattern in mammals, H3K4me3, H3K9ac, and H3K36me3 were common marks in the active wheat genes. There was a small difference in terms of the H3K4me3 pattern, which is highly enriched in the transcription start site (TSS) of mammals, but extends into the gene body regions in wheat (Additional file 1: Figure S1). Fourth, a considerable abundance of the H3K4me1 mark is a typical feature of enhancers in mammals, whereas H3K4me1 preferentially marks gene body regions in wheat and other plant species [16, 34] (Additional file 1: Figure S1). These observations imply that the regulatory role of this mark evolved independently in plants and mammals. Finally, in mammals, enhancers marked by H3K27me3 are largely in a poised state [35]. Similarly, we observed that in wheat, the features of chromatin state 9 (Fig. 3a), accounting for 0.8% of the whole genome sequence, were similar to those of chromatin state 5, which corresponds to the highly conserved regulatory regions. Both states were similar regarding chromatin accessibility, H3K9ac marks, CGI coverage, and conservation scores (Fig. 3). The only difference was that chromatin state 9 had a large abundance of H3K27me3. Whether this state represents the repressed regulatory elements in wheat seedlings will need to be experimentally verified. Considering that enhancers generally exhibit high tissue specificity, future studies including diverse tissues under various experimental conditions will lead to a more comprehensive characterization of the dynamic roles of these regulatory elements influencing transcription.

Our functional validation of the selected regions suggested that about 50% of the regions were associated with high luciferase expression in the reporter assay. This ratio is somewhat lower than the positive ratio (7/9 = 78%) for the predicted human enhancers based on a similar computational method. It is worth noting that 50% positive ratio in the present study is a stringent estimation which did not take in to account regions showing active enhancer activity in one or two of the three experimental replicates, in order to present the most reliable enhancer-like regions validated. It is possible that the active cis-regulatory regions predicted herein may require additional temporal or spatial trans-acting factors for the enhancer function. Additionally, considering the large bread wheat genome and complex interactions between the three sub-genomes, it is possible that there are abundant inter- and long distance intra-chromosomal interactions that may be coordinated to regulate transcriptional activity. The cis-elements predicted in the present study provide good candidates for future studies of the transcriptional regulation by large-scale chromosomal interactions. Considering the dynamics and specificity of enhancers, future studies involving diverse tissues under various experimental conditions are needed to more comprehensively elucidate the roles of these regulatory elements influencing transcription.

## Conclusion

The regulatory elements detected based on the comprehensive profiling of chromatin structures are valuable resources for the plant community. The findings and approaches described herein provide insightful clues for future investigations of the evolution of gene regulation during wheat polyploidization events.

## Methods

### Plant materials and growth conditions

The bread wheat (*Triticum aestivum*) cultivar “Chinese Spring” was analyzed in this study. Seeds were sterilized by a 10-min incubation in 30% H_2_O_2_ and then thoroughly washed five times with distilled water. The sterilized seeds were germinated in water for 3 days at 22 °C, after which the germinated seeds with residual endosperm were transferred to soil. After a 9-day incubation under long-day conditions, the aboveground parts were harvested. The harvested samples were either frozen in liquid nitrogen for an RNA isolation step or vacuum-infiltrated with a formaldehyde cross-linking solution for a ChIP assay.

### ChIP and RNA sample preparation and sequencing

A ChIP assay was completed as previously described [36], with antibodies specific for H3 trimethyl-Lys 27 (Upstate, USA), H3 trimethyl-Lys 4 (Abcam), H3 trimethyl-Lys 36 (Abcam), H3 dimethyl-Lys 9 (Abcam), H3 monomethyl-Lys 4 (Upstate), H3 acetyl-Lys 27 (Upstate), and H3 acetyl-Lys 9 (Upstate). For each ChIP-sequencing assay, approximately 30 seedlings were pooled together and ground to a powder. More than 10 ng ChIP DNA, 2 μg mRNA and 2 μg total RNA (rRNA depleted) were used to prepare each sequencing sample. Libraries were constructed and sequenced by Genenergy Biotechnology Co. Ltd. (Shanghai, China). For total RNA-seq, the strand-specific library was constructed as previously described [37]. The libraries were sequenced with the HiSeq 2000/2500 system (Illumina) to produce 150-bp paired-end reads. The sample preparation, library construction, and sequencing were completed with two biological replicates. Sequencing data for the replicates had relatively high Pearson correlation coefficients (Additional file 1: Figure S6). The ChIP-sequencing data were highly correlated with the data from a recently published study [13], which involved a ChIP-sequencing analysis of H3K27me3, H3K4me3, and H3K9ac (Additional file 1: Figure S7).

### DNase-sequencing library preparation and sequencing

The harvested seedlings used to prepare ChIP-sequencing samples were also subjected to a DNase I treatment. Specifically, to prepare DNase-sequencing libraries, approximately 20 seedlings were fixed with 1% formaldehyde in HEPES buffer (50 mM HEPES, 1 mM EDTA, pH 8.0, 0.1 M NaCl, and 1 mM PMSF). The fixed seedlings were ground to a fine powder in liquid nitrogen. Wheat nuclei were extracted with H1B buffer (20 mM Tris-HCl, pH 8.0, 50 mM EDTA, 5 mM spermidine, 0.15 mM spermine, 40% glycerol, and 0.1% mercaptoethanol). The extracted nuclei were purified with H1B buffer supplemented with 0.5% Triton X-100. The purified nuclei were washed once with RSB buffer (10 mM Tris-HCl, pH 7.4, 10 mM NaCl, and 3 mM MgCl_2_). The pelleted nuclei were resuspended with 2 ml RSB buffer and then divided equally into five 1.5-ml Eppendorf tubes. The aliquoted nuclei were digested with DNase I (0, 0.01, 0.03, 0.05, and 0.08 units). The resulting digested nuclei were extracted using 1 volume of phenol, phenol:chloroform, and chloroform, after which the DNA from each digestion was resuspended in 2 volumes of cold ethanol and then pelleted. A DNase-sequencing library was prepared from 0.03 U DNase I-digested nuclei. Approximately 2 μg DNA was separated by 1.5% agarose gel electrophoresis, and DNA fragments (50–300 bp) were cut and purified to prepare the DNase-sequencing library with the NEBNext® Ultra™ II DNA Library Prep Kit for Illumina (NEB). Two biological replicates of the libraries were prepared. The quality of the final DNase-sequencing libraries was checked, after which the libraries were sequenced with the 150-bp paired-end mode of the Illumina NovaSeq platform.

### Processing of sequencing data

Sequencing reads were cleaned with the Trim Galore (version 0.4.4), Trimmomatic (version 0.36) [38], and Sickle programs, which eliminated bases with low-quality scores (< 25) and irregular GC contents, sequencing adapters, and short reads. The remaining clean reads were mapped to the International Wheat Genome Sequencing Consortium (IWGSC) reference sequence (version 1.0) with the Burrows-Wheeler Aligner-MEM (version 0.7.5a-r405) [39] for the DNase-sequencing and ChIP-sequencing data. To ensure homolog specificity and accuracy for subsequent analyses, the mapping results were further stringently filtered as follows: (1) remove reads with mapping quality (*Q*) < 20 [*Q* = − 10 log_10_(*p*), where *p* is an estimate of the probability that the alignment does not correspond to the read’s true point of origin]. Additionally, *Q* > 20 ensures the reads are mostly uniquely mapped; (2) remove all supplementary alignments with SAM flag = 2048; (3) keep only the correctly mapped read pairs; and (4) remove duplicated reads mapped to exactly the same position because they were considered to be artifacts caused by the PCR during the library construction step. The HISAT2 program (version 2.1.0) [40] was used for mapping the RNA-sequencing reads to the reference sequences and gene models from the IWGSC RefSeq genome assembly (version 1.0). High-confidence genes from this gene model version were used throughout this study. The predicted new transcripts were also compared to the low-confidence genes listed in Additional file 2: Table S3. The ChIP-sequencing data were also analyzed with MACS (version 1.3.7) [41] to identify read-enriched regions (peaks) based on the following criteria: *P* value < 1e−50 and fold-change > 32. Target genes were defined as genes with a peak within or near the gene body (± 2 kb). To quantify gene expression levels, the featureCount program of the Subread package (version 1.5.3) [42] was used to determine the RNA-seq read density of the high-confidence genes in the IWGSC RefSeq genome assembly (version 1.0). To compare expression levels across samples and genes, the RNA-seq read density of each gene was normalized based on the exon length in the gene and the sequencing depth [i.e., fragments per kilobase of exon model per million mapped reads (FPKM)]. To quantify histone markers and DHS signals across genes for the illustration prepared with Integrative Genomics Viewer [43], the number of reads in each position was normalized against the total number of reads (reads per million mapped reads).

### Bisulfite sequencing and data analysis

Bisulfite sequencing samples were prepared with 2.2 μg DNA extracted from the harvested seedlings that were also used to prepare the ChIP-sequencing and DNase-sequencing samples. The bisulfite sequencing libraries were constructed and the subsequent deep sequencing was completed by Genenergy Biotechnology Co. Ltd. (Shanghai, China). The libraries were sequenced with the HiSeq 3000 system (Illumina) to produce 150-bp paired-end reads, which were cleaned as described above. The clean reads were then aligned to the IWGSC reference sequence (version 1.0) with the default settings of the Bismark program (version 0.19.0) [44]. The default settings were strict, with only the best unique alignments reported, and all non-unique alignments were removed [44]. Thus, we applied only two additional filtering steps, namely the removal of reads with a mapping quality < 20, followed by the removal of PCR duplicates with the deduplicate_bismark implemented in the Bismark program (version 0.19.0).

The extent of the cytosine methylation was determined with the bismark_methylation_extractor implemented in the Bismark program (version 0.19.0). Next, the methylation ratio of a cytosine was calculated as the number of mCs divided by the number of reads covering the position. Bases covered by fewer than three reads were considered low-confidence positions whose methylation ratios were not recorded.

### Definition of triad genes

High-confidence gene models from the IWGSC RefSeq genome assembly (version 1.0) were used for defining triad genes. OrthoFinder [45] was applied to detect orthogroups for all homologous genes across the three sub-genomes. A total of 18,313 orthogroups, with only one gene copy in each sub-genome, were defined as triads. We considered a triad was expressed when the sum of the FPKM of homologs from three sub-genomes > 1. Thus, 12,669 expressed triads were detected.

### Detection of CpG islands

The CGIs were detected with the R package makeCGI [46], which involves two HMMs for the GC content and the ratio of observed versus expected CpGs. To determine the posterior probability cutoff, we assessed the sensitivity, which was defined as the percentage of TSSs covered by a CGI given the observation that CGIs were enriched surrounding TSSs (Additional file 1: Figure S5, see below). The receiver operating characteristic (ROC)-like plot in Additional file 1: Figure S5 presents the fraction of TSSs covered by a CGI (used as a measure of sensitivity) versus the total length of different CGIs (used as a measure of specificity) defined with posterior probability cutoffs ranging from 0.5 to 0.9995. We chose 0.99 as a cutoff value because it is associated with the inflection point of the ROC curve. With 0.99 as a cutoff, we determined that 43.4% of the TSSs were covered by a CGI, which is comparable to the ratios in humans and mice (50%–56%) [46]. A further comparison with the proportion of TSSs covered by random regions with the same number and length distribution of a given CGI suggested that CGIs are apparently enriched at TSSs in wheat (Additional file 1: Figure S5).

### Identification of newly formed genes in hexaploid wheat

The MUMmer 4.0 program [47] was applied to compare the genome sequences of hexaploid wheat *T. aestivum* (AABBDD, IWGSC version 1.0) and the progenitors, including *Triticum turgidum* (AABB, WEWSeq version 1.0), *Triticum urartu* (AA, Tu2.0), and *Aegilops tauschii* (DD, ASM34733 version 2). Collinear regions identified by MUMmer with a sequence identity > 90% were defined as “similar regions”. To increase the stringency of the definition of old and young genes, OrthoFinder [45] was used to detect orthologous genes between hexaploid wheat and the progenitors. Orthologous genes within similar regions (sequence identity > 90%) in hexaploid wheat and the progenitors were defined as “old” genes (100,166 genes), whereas hexaploid wheat genes outside these similar regions and with no orthologues in wild progenitors were defined as “young” genes (10,624 genes). All gene models were downloaded from published annotated reference genomes. The *T. urartu* (AA) gene model was downloaded from http://www.mbkbase.org/Tu/ [48], whereas the gene model of *A. tauschii* (DD) was obtained from a published study [49] and the gene model of *T. turgidum* (AABB) (WEWseq_PGSB_v1) was downloaded from https://www.dropbox.com/sh/3dm05grokhl0nbv/AADLrcn1iV48S1pTRpd0PJm4a/Annotation?dl=0&subfolder_nav_tracking=1 [50].

### Definition of homolog expression and epigenetic-binding bias categories

A previously described ternary plot-based method was applied for defining epigenetic binding and expression patterns of triads [13]. For each homologous gene from one sub-genome, the intensities of expression or histone marks in the promoter region were normalized against the total densities within the triad to represent the relative expression or binding. Next, Euclidean distances of each gene along the three angles of the ternary plot were determined based on the fraction of the reads mapped to the given gene triad. The significance of the differential expression between homologs was determined using the DEseq program [51], with the cutoff set to *P* < 0.05. Genic regions with a significantly higher level of binding in the A sub-genome than in the other two sub-genomes were defined as A-dominant. Genic regions with a significantly lower level of binding in the A sub-genome than in the other two sub-genomes were defined as A-suppressed. Other regions with no significant binding differences among sub-genomes were defined as balanced regions.

### Detection of chromatin states

To assess the combinatorial patterns of seven chromatin marks, we applied a previously developed machine-learning method based on a multivariate HMM, ChromHMM (version 1.17) [52], which defined distinct chromatin states representing various combinatorial presence/absence patterns for multiple marks.

### Calculation of the sequence conservation score

We completed a pair-wise comparison of the genome sequences from *T. aestivum* (AABBDD sub-genome, IWGSC version 1.0), *T. turgidum* (AABB sub-genome, WEWSeq version 1.0), *T. urartu* (AA sub-genome, Tu2.0), and *A. tauschii* (DD sub-genome, ASM34733 version 2) with the nucmer tool implemented in the MUMmer package [47]. The minimum sequence identity was set to 90 and each sub-genome was treated as an individual genome. Next, ROAST [53] was used to integrate pair-wise sequence alignments into a multiple sequence alignment. The multiple sequence alignment and tree data were fitted by PhyloFit, after which the conservation score was calculated with phastCons from the PHAST package [54].

### Characterization of the coding potential

The protein coding ability of newly predicted transcripts was assessed using coding potential calculator (CPC) [55]. For 3327 transcripts belonged to neither high-confidence nor low-confidence gene models, 3138 (94%) have the coding potential score lower than 0.5 (Additional file 2: Table S3).

### Calculation of the similarity index of chromatin signatures between sub-genomes

To quantify the similarity of chromatin states between the collinear regions of sub-genomes, we calculated the Jaccard similarity index [56] of the 15 chromatin states for a pair-wise comparison between sub-genomes. The Jaccard similarity index was defined as $\frac{A\bigcap B}{A\bigcup B}$, where *A* is the number of sequences in a certain chromatin state of one sub-genome and *B* refers to the number of sequences in the corresponding state of another sub-genome.

### Detection of transcription factor-binding motifs

To detect enriched transcription factor-binding motifs in the proximal promoter and enhancer-like regions, we first detected the proximal (3 kb upstream of the nearest TSS) and distal regions corresponding to state 5, which displayed typical epigenetic features corresponding to cis-regulatory elements, and are highly conserved across sub-genomes, thus accurately representing regulatory activities. A total of 46,834 proximal (promoter) and 23,563 distal (enhancer-like) regions in state 5 were detected (Additional file 2: Table S4). For the subsequent motif analysis, we downloaded the position weight matrices of 501 plant motifs from the JASPAR database [57]. The motifs were then scanned against the regions detected using the Find Individual Motif Occurrences program [58] of the MEME software toolkit (version 5.0.2). The number of motifs in the promoter and enhancer-like regions was normalized against the total number of scanned regions.

### Cloning

The minimal 35S promoter (− 50 to − 2 bp) derived from the CaMV 35S promoter was ligated into the pMY155 vector (from Dr. Lin Xu, SIPPE). The sequences of the predicted enhancer-like elements were amplified from the Chinese Spring genomic DNA (primers are listed in Additional file 2: Table S5) and were cloned into pMY155-mini35S. The pMY155-mini35S construct with or without the predicted enhancer-like elements were transferred into *Agrobacterium tumefaciens* strain GV3101 cells.

### Agroinfiltration of *Nicotiana benthamiana* leaves

*Nicotiana benthamiana* plants were grown in a greenhouse for 3–4 weeks before the agroinfiltration step. To suppress gene silencing, *A. tumefaciens* cells expressing the p19 protein of the tomato bushy stunt virus [59] were used in the co-infiltration procedure. Overnight cultures of *A. tumefaciens* were used to inoculate a 10-fold larger volume of fresh YEB liquid medium. The cells were cultured for another 4–6 h, pelleted, resuspended with infiltration buffer (10 mM MgCl_2_, 10 mM MES, pH 5.7, and 200 μM acetosyringone), and incubated for another 2 h. A mixture of *A. tumefaciens* strains containing reporter plasmids and the p19 strain at an optical density (600 nm) ratio of 0.8:0.8 was prepared for the co-infiltration of the abaxial side of *N. benthamiana* leaves with a needleless syringe. A bioluminescence scan of at least three leaves was completed 2 days after the infiltration. Experiments were repeated at least three times for each predicted enhancer-like element.

### Dual luciferase assay

Transformed leaves were sprayed with 1 mM D-luciferin solution containing 0.01% Triton X-100. Samples were then incubated in darkness for 2 min. The bioluminescence of whole leaves was visualized with the ImageQuant LAS 4000 system (GE Life Sciences) to record the photon emission over a 3-min interval. Image J software (version 1.52) was used to measure the luciferase signal and create image overlays. The dual luciferase assay was completed with the BioTek Synergy 2 microplate reader and the Dual-Luciferase Reporter Assay System (Promega) according to the manufacturer’s instructions. The relative ratio of firefly luciferase: Renilla luciferase activities were calculated, and the average of three independent biological replicates was recorded for each plasmid.

## Additional files

Additional file 1: Supplementary figures (DOCX 2144 kb)

Additional file 2: Supplementary tables (XLSX 15730 kb)

Additional file 3: Review history (DOCX 1422 kb)

## Data Availability

The ChIP-sequencing and RNA-sequencing data were deposited in the Gene Expression Omnibus database [60]. Tracks for all sequencing data, chromatin states can be visualized through our local genome browser (http://bioinfo.sibs.ac.cn/cs_epigenome). RNA-seq data for seven tissues of bread wheat, including root, spike, stem, grain, leaf, pistil, and stamen, were published previously [61]. For demonstration of data reproducibility, both ChIP-seq data (H3K27me3, H3K4me3, H3K9ac, and H3K36me3) and whole-genome bisulfite sequencing data were compared with the data published recently [62].

## Abbreviations

CGI: CpG island
ChIP-sequencing: Chromatin immunoprecipitation followed by high-throughput sequencing
DHS: DNase I-hypersensitive site
NB-LRR: Nucleotide-binding site leucine-rich repeat
PcG: Polycomb group
TE: Transposable element
TES: Transcription end site
TSS: Transcription start site
