# Supplementary material for: The bread wheat epigenomic map reveals distinct chromatin architectural and evolutionary features of functional genetic elements
Source: Genome Biol. 2019 Jul 15;20:139. doi: 10.1186/s13059-019-1746-8 (PMC6628505; doi:10.1186/s13059-019-1746-8)
Supplement: Supplementary file 1 — Supplementary figures (DOCX 2144 kb) [file 13059_2019_1746_MOESM1_ESM.docx]

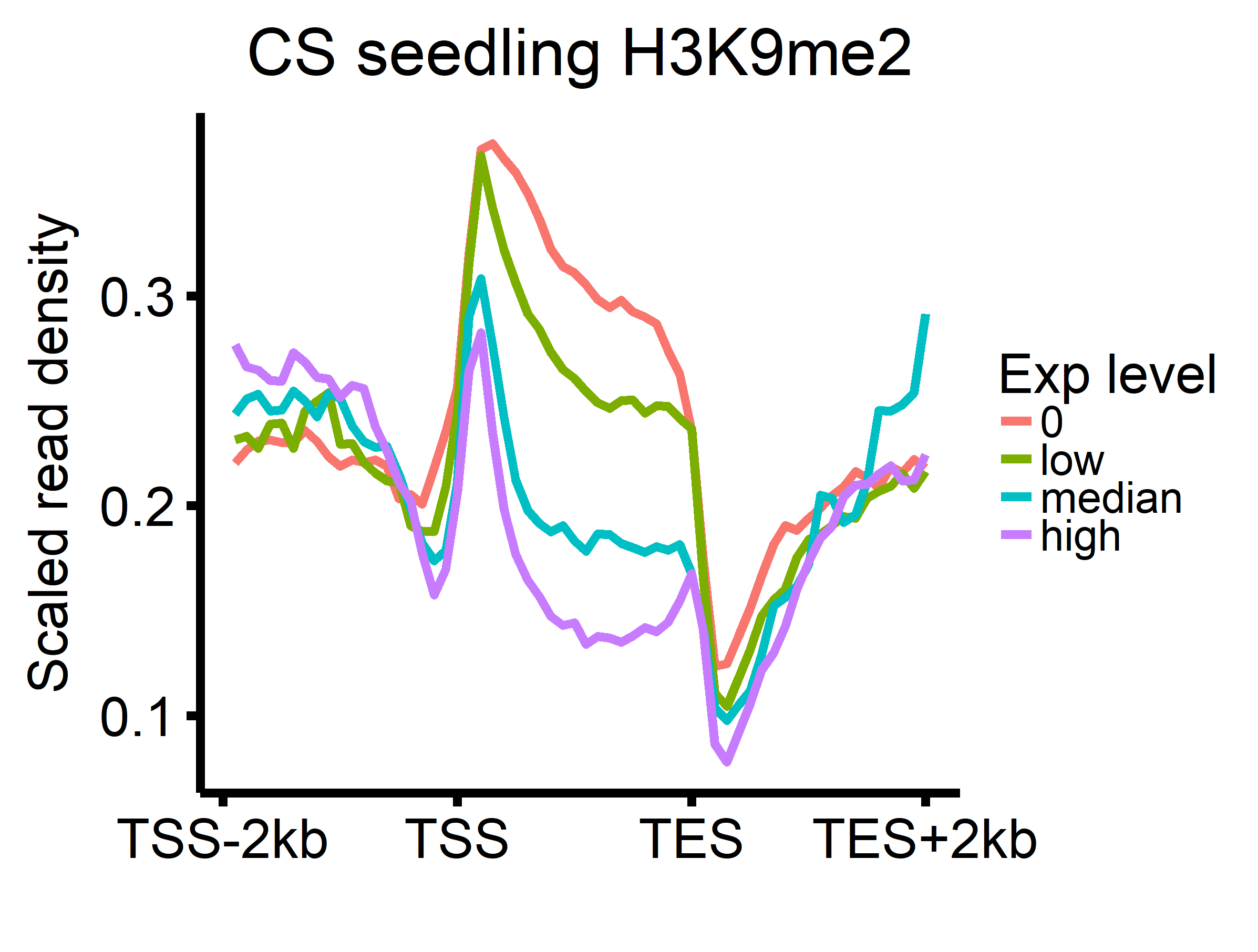

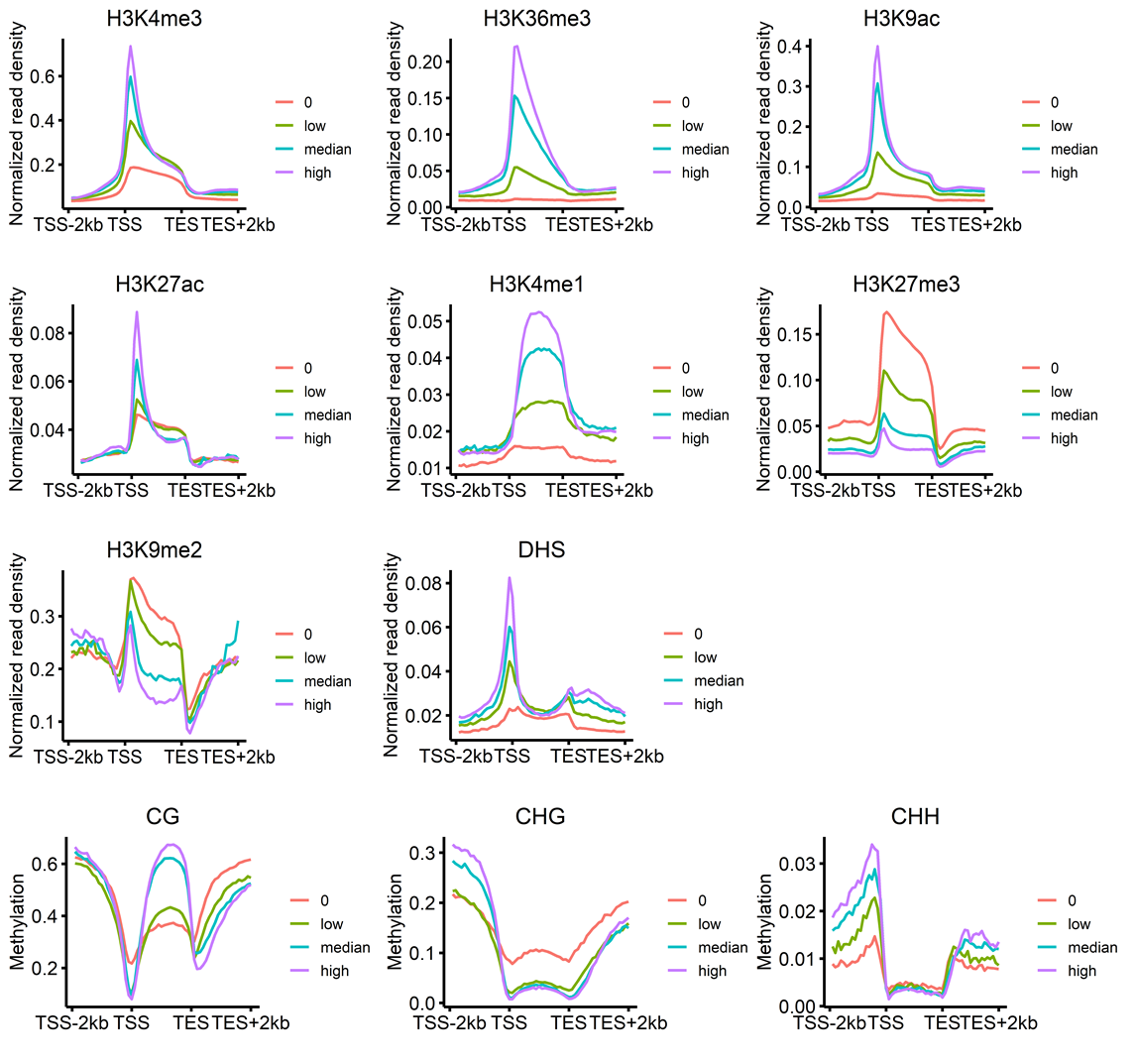


**Fig S1.** Average profiles of the read density of seven histone marks, the DHS, and DNA methylation levels surrounding differentially expressed genes. Coordinates of high-confidence gene models from the IWGSC RefSeq genome assembly (version 1.0) were used. Regions from 2 kb upstream and 2 kb downstream of gene body regions were divided into 60 tiles (20 upstream tiles, 20 downstream tiles, and 20 gene body tiles), and the average methylation ratio in each tile was recorded and plotted.

**
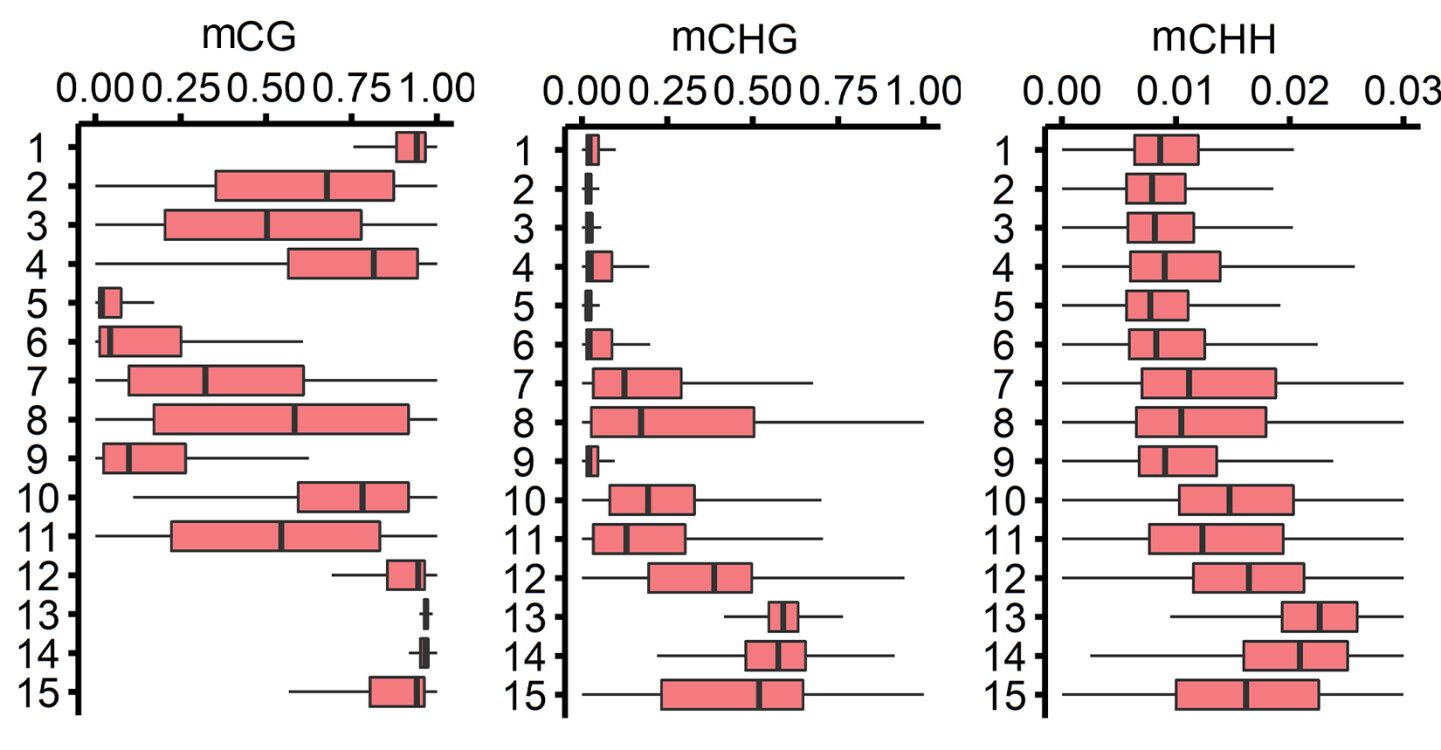
**

**Fig S2.** Boxplots presenting the distribution of the DNA methylation ratio of each chromatin state in three sequence contexts. Recently published bisulfite sequencing data were used (DOI: 10.1126/science.aar6089). The results are consistent with those obtained with the data generated in the present study.


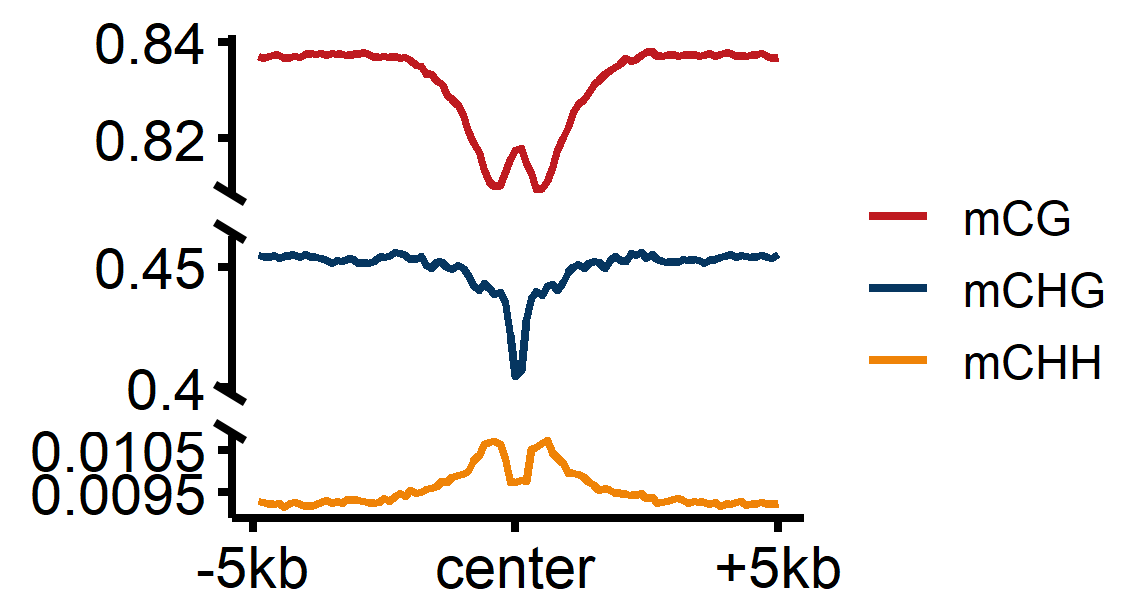


**Fig S3.** Average profiles of the DNA methylation ratio in three contexts surrounding the centre position of a wheat CGI.


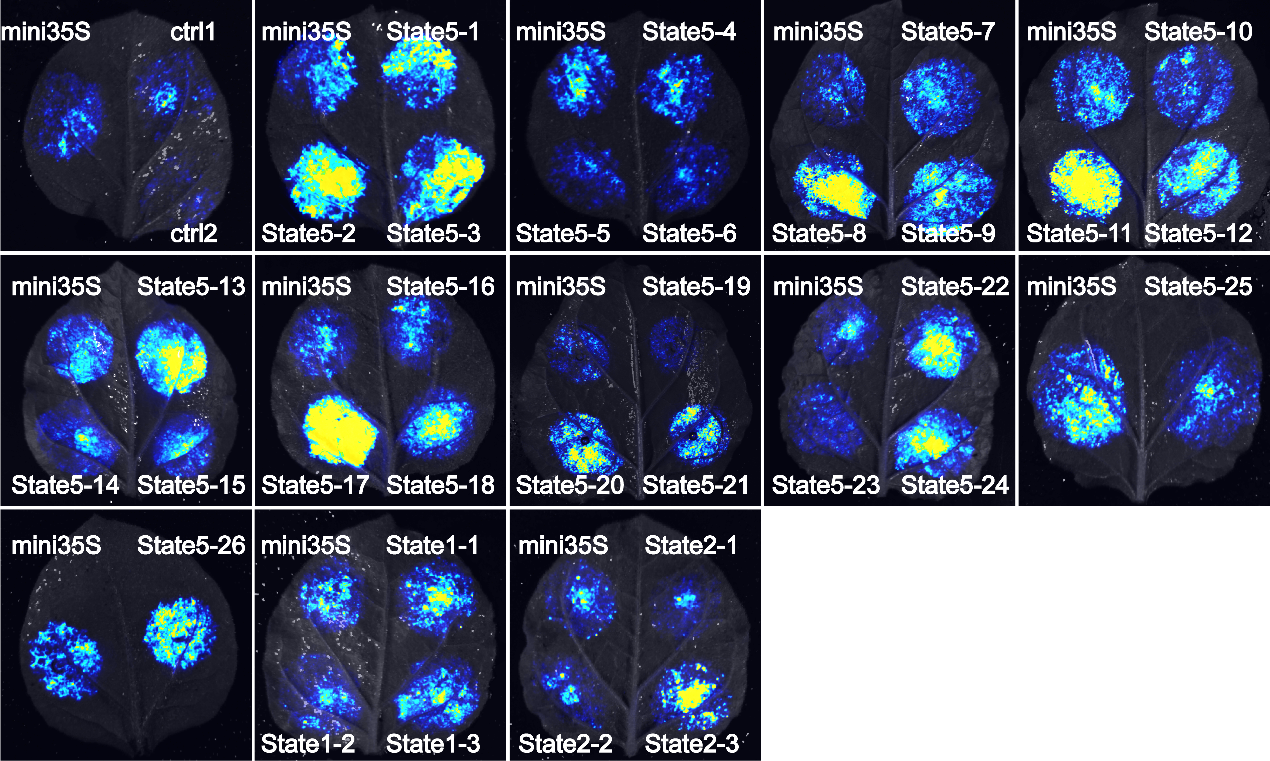


**Fig S4.** Validation of the enhancer activity of 26 predicted distal regulatory elements based on a luciferase reporter assay. The coordinates of the elements are listed in Supplementary Table 5.


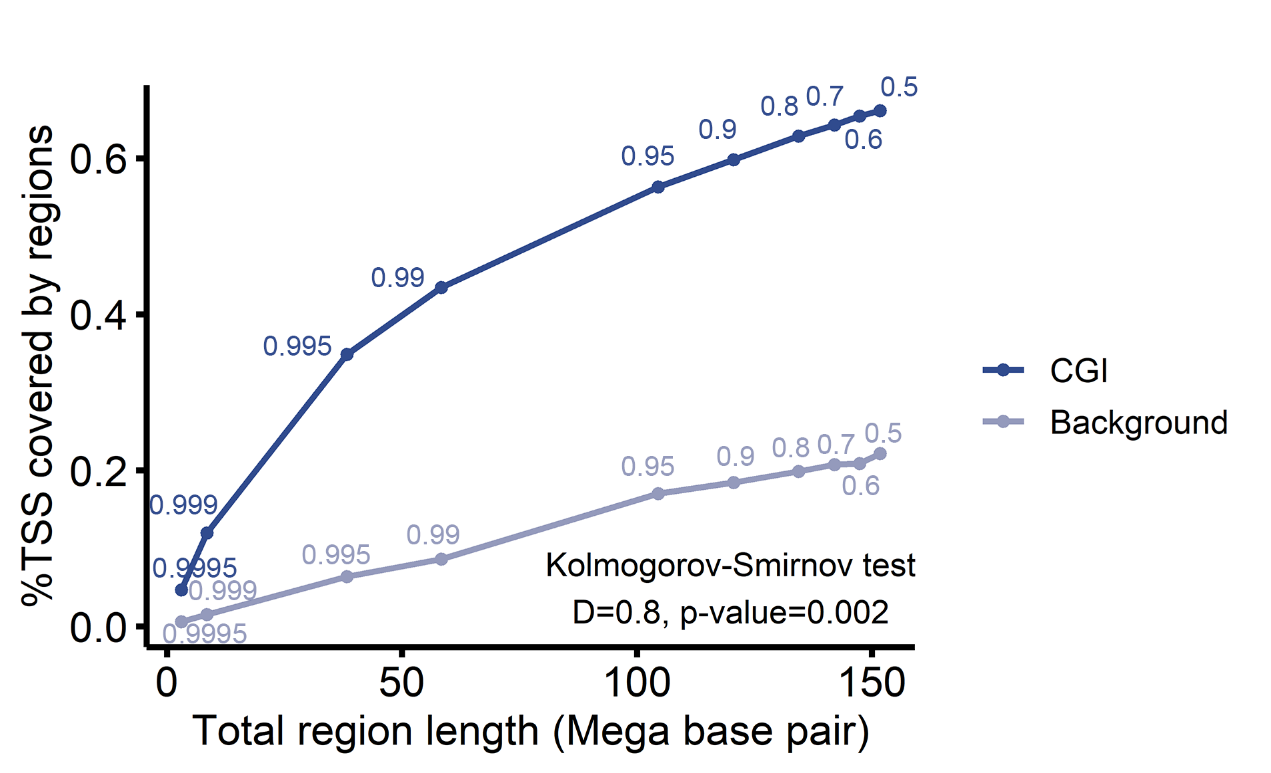


**Fig S5.** Receiver operating characteristic-like plot presenting the percentage of TSSs covered by a CGI (used as a measure of sensitivity) versus the total length of different CGIs (used as a measure of specificity) defined with posterior probability cutoffs ranging from 0.5 to 0.9995. The chromosome 1A sequence was used for analysing sensitivity.


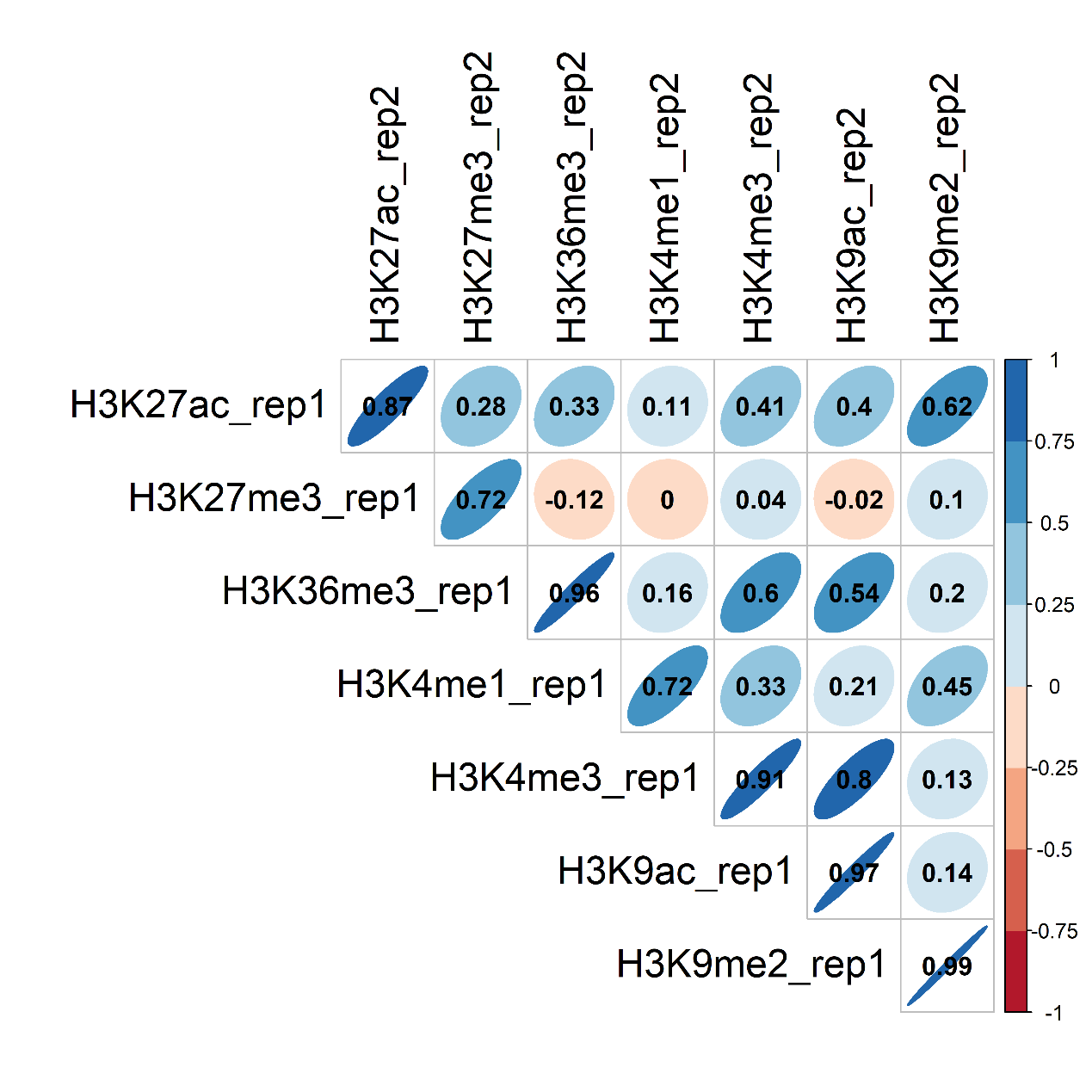


**Fig S6.** Plot of the correlation between the data for biological replicates. Genomic regions were divided into consecutive 500-bp bins, and the bins overlapping the peak region of any marks were selected. Read densities in the selected bins were recorded for calculating sample correlation coefficients.


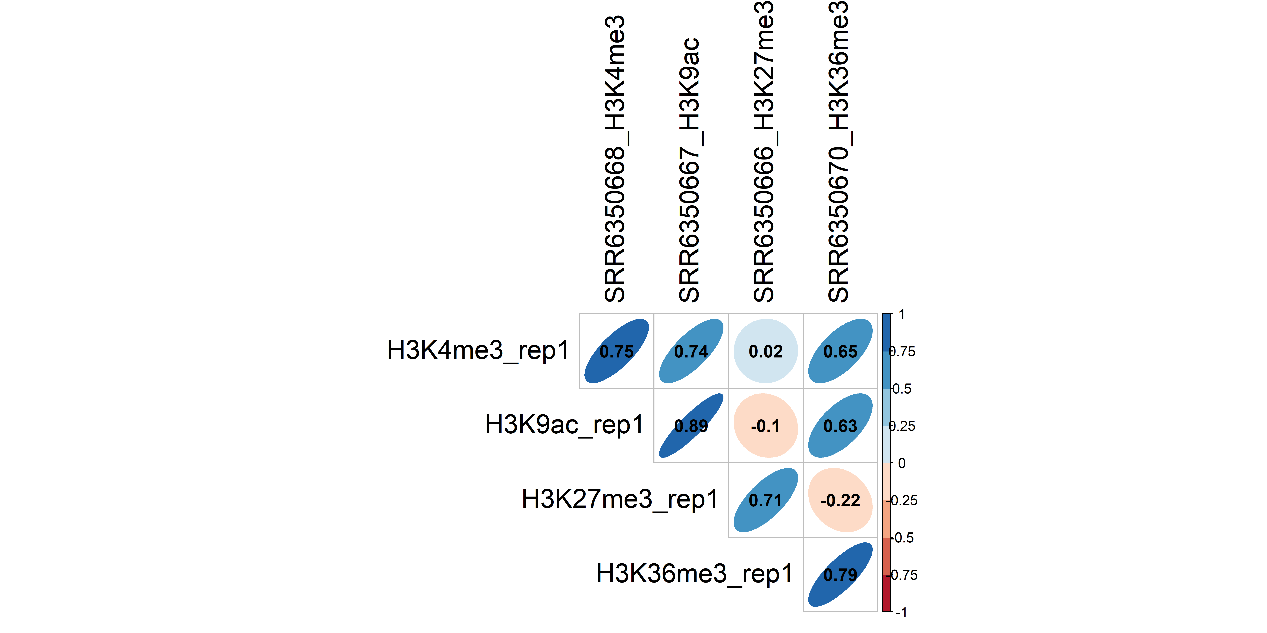


**Fig S7.** Plot of the correlation between ChIP-seq data from the present study and recently published data (doi: 10.1126/science.aar6089). Genomic regions were divided into consecutive 500-bp bins, and the bins overlapping the peak region of any marks were selected. Read densities in the selected bins were recorded for calculating sample correlation coefficients.
