# Supplementary material for: The bread wheat epigenomic map reveals distinct chromatin architectural and evolutionary features of functional genetic elements
Source: Genome Biol. 2019 Jul 15;20:139. doi: 10.1186/s13059-019-1746-8 (PMC6628505; doi:10.1186/s13059-019-1746-8)
Supplement: Supplementary file 3 — Review history (DOCX 1422 kb) [file 13059_2019_1746_MOESM3_ESM.docx]

Review History

**First round of review**

**Comments to author and authors’ point-by-point responses to the reviewers’ comments:**

**Reviewer 1**

Li et al. profiled chromatin architecture, epigenomic marks and gene expression in the bread wheat genome and predicted regulatory elements based on grouping chromatin signatures. I'd like to recommend a significant re-focus of the manuscript that takes into account what is already known, analyses and highlights any differences and the additions of the authors' work. In particular, the abstract statement 'We revealed that evolutionary selection on gene regulation is targeted at the level of both sequence and chromatin features' doesn't seem to be discussed or justified anywhere in the manuscript at the moment but would be interesting. The abstract conclusion that this is the first systematic epigenomic map for wheat is just not the case, I'm afraid, and, after reading the manuscript, I don't find anything more systematic than what has gone before or understand 'for functional annotation', but would be interested if that were possible. More disappointingly, I don't agree that the manuscript provides new insights into connections between chromatin modifications and transcriptional regulatory activities yet.

*Response:*

*- Thank you for the comments. We agree that this study is not “the first epigenomic map for wheat”, and have changed this sentence in the Abstract to, “We herein present a systematic epigenomic map for the annotation of cis-regulatory elements in the bread wheat genome...” (page 3, line 23).*

*- We agree that the statement, “This manuscript provides new insights into connections between chromatin modifications and transcriptional regulatory activities”, is not appropriate here. We have revised the sentence to, “...provides new insights into the connections between chromatin modifications and cis-regulatory activities in allohexaploid wheat.” (page 3, lines 26).*

*- Regarding the statement, “We revealed that evolutionary selection on gene regulation is targeted at the level of both sequence and chromatin features”, we determined that in addition to conserved sequences (Fig. 3f), regulatory states, especially state 5, were highly similar across the three sub-genomes regarding epigenetic features (Fig. 4 in the revised manuscript), indicating that cis-elements from this state underwent a purifying selection at the sequence and epigenetic activity levels. To clarify this point, we made the following three revisions:*

*1) We revised a sentence in the Abstract (page 3, lines 18–19) to, “A comparison across sub-genomes revealed that evolutionary selection on gene regulation is targeted at the sequence and chromatin feature levels.”*

*2) We converted Fig. 4a in the previous manuscript to a separate figure, which also includes an IGV screen shot (Fig. 4b) to illustrate the similarity of the epigenetic activities of collinear intergenic regions across three sub-genomes. We also describe the data presented in Figure 4 in the Results section. Please refer to Fig. 4, page 11, the 2^nd^ paragraph in the Results section. 3) We now discuss this issue. Please refer to page 13, lines 10–13 in the Discussion section.*

Major points:

The rationale for much of the work and what it adds to knowledge is not clear, at least to me as a plant biologist:

1. The authors briefly reference the 2018 publication Ramirez-Gonzalez et al (reference 12) but don't discuss that paper at all. That report included a more comprehensive dataset and I think, although I'm not sure, Li et al. have used some of those data in their analyses (e.g. the tissue-specific expression Table S1 and Fig. 1e) but in other cases have used their own dataset. The conclusions as they regard chromatin states, methylation and chromatin marks, homeolog suppression/ dominance and gene expression are similar to those of Ramirez-Gonzalez et al. so, although that gives some degree of confidence in the robustness and reproducibility of the results, no new findings, insights or biological significance are added. In most cases, the Ramirez-Gonzalez dataset is markedly better than the one used here, and I was left wondering whether Li et al. might be better to re-analyse the Ramirez-Gonzalez et al. data to answer their different questions. For example, the choice of accessions and sequencing data from diploid and tetraploid genomes in Ramirez-Gonzalez et al. would improve the calling of old versus young genes here.

*Response: Thank you for pointing this out. We apologize for the over-simplified description of the study by Ramirez-Gonzalez et al. (2018) in the submitted manuscript. We re-wrote the Background section and now describe in detail the findings of Ramirez-Gonzalez et al. (pages 4–5, the second paragraph of the Background section). Additionally, we now indicate which of our results confirm those of Ramirez-Gonzalez et al. and which are new findings (page 6, lines 26–27 and page 8, the first paragraph).*

*Regarding the dataset from Ramirez-Gonzalez et al., we performed the following three analyses:*

*1) Supplementary Figure 7 presents the high correlation between our data and those of Ramirez-Gonzalez et al.*

*2) Because the DNA methylation sequencing depth is greater for the data of Ramirez-Gonzalez et al. than for our dataset, we used the DNA methylation data from Ramirez-Gonzalez et al. to validate our major conclusions in Supplementary Figure 2.*

*3) Regarding the definition of old and young genes, we used the same gene accessions and sequences of diploid and tetraploid genomes as those used by Ramirez-Gonzalez et al. We now define old and young genes in the Methods section. Please refer to page 20, the 2nd paragraph.*

2. The difference here in approach and interpretation compared with the Ramirez-Gonzalez paper is, broadly, the comparison with mammalian genomes and the prediction of regulators. The first case is the prediction of CpG islands. As I understand it, CpG islands and shores in mammalian and invertebrate genomes may be associated with functionally relevant regulatory regions of differential methylation. As the authors rightly say here, (p. 11) whether they have any functional relevance in plants is unknown and there were no functional studies carried out here. The authors report they found a relatively high abundance of CGIs in chromatin states 5-7 where DNA methylation is low and that this was suggestive of a similar relevance and conservation as in mammalian genomes. Of course, with these large datasets it is difficult to be sure of groupings, but 'relative' is not well-defined.

For example, chromatin state 7 looks in Fig.3 to have a very similar proportion of CGIs to chromatin state 10 which has high methylCG.

Chromatin state 13 looks as though it has a similar proportion of CGIs to chromatin state 5 (the highest?) and is the largest chromatin state in the genome, with high methylation in all contexts, high H3K9me2 and low DHS density. From the results as presented, I don't think the authors can justify the conclusion that CGIs are especially enriched in regulatory regions of the wheat genome or depleted in methylation, or the suggestion that they may be functionally conserved between plants and mammals. Without functional studies, I'm sorry but I didn't find these results convincing or that they added any new insight.

*Response: Thank you for pointing this out. In addition to states 5–7, CGI proportions were high for states 9, 10, and 13 (Fig. 3h). States 9 and 10 exhibited high H3K27me3 activity, which is consistent with a published report indicating that CGIs in mammals may be repressed by H3K27me3 catalysed by the PcG complex (doi:10.1101/gad.2037511). However, the CGI enrichment in state 13 (i.e., H3K9me2 marked regions) differs from the results in a human study, which revealed that CGI+ regions are largely lacking in H3K9me2/3 (doi.org/10.1093/nar/gky147). We now describe and discuss the association between CGIs and epigenetic features in wheat in greater detail (page 14, lines 4–15 in the Discussion section).*

3. The one, new addition of this paper is the prediction of enhancer-like elements, distal enhancer elements and relationship with chromatin profiles (promoter analysis including TE occupancy, transcription factor prediction and analysis of expression networks related to transcription factor binding with tissues and treatments are reported extensively in Ramirez-Gonzalez et al but not discussed here). Li et al. present a functional validation of their findings for enhancer-like elements and emphasize how these predictions can be useful in subsequent epi/genomic work with wheat. However, I think there is more work or correction necessary to ensure the quality and robustness of the reported results.

Firstly, there is what I suppose is just a mistake in table S5 where enhancers 2, 3 and 6 are marked as positive whereas, from Fig. 4, I suppose this should be enhancers 1, 2 and 3. If it's not a mistake, some explanation would be required.

*Response: Thank you for pointing out the mistake in Supplementary Table 5, which was based on the original order of the experiments. To prepare Figure 4, we repeated the experiment and included the robustly positive enhancers together. Our mistake has been corrected in the revised manuscript (please refer to Supplementary Table 5).*

- Secondly, the putative enhancers are all chosen from one region differently distal to one gene and so it is difficult to conclude that this validates a genome-wide pattern. Thirdly, they are all chosen from one major chromatin state and, to validate the connection or relationship properly, it would be necessary to compare with at least one other chromatin state and preferably more.

*Response: The regions were chosen from regions related to different genes (Supplementary Table 5). We agree that relatively few regions were included in the experimental validation in the submitted manuscript. We have added 20 additional regions from state 5, and another six regions from states 1 and 2, including exon and intron regions, for the experimental validation. Please refer to page 15, the second paragraph, Figure 6, and Supplementary Table 5.*

- Finally, the choice and significance of proximal versus distal regions of promoters and enhancers seems inconsistent through the manuscript and Fig. 4 f and g scales are unclear. Is the scale 0 - 1.2 fold and is this significant enrichment?

*Response:*

*- Sorry for the confusion due to insufficient details. The scales in Figure 4f and g were normalized scales. Figure 4f is now Figure 5e in the revised manuscript. The colour bar in Figure 4f of the submitted manuscript belonged to the heatmap, which was based on the total number of motifs normalized against the number of promoter or enhancer-like regions, from which the enrichment score was calculated and plotted (the bar plot to the left of the heatmap). To avoid any confusion, we removed the heatmap in Figure 4f of the submitted manuscript, but kept the bar plot to present the difference in the motif enrichment score between promoter and enhancer like regions (Fig. 5e in the revised version). We added a detailed description to the Figure 5e legend. The enrichment is around 1.4- to 2-fold (log-scaled approximately 0.5–1) as shown on the x-axis. The font size in the previous version was too small to be seen. Accordingly, we increased the font size of the x-axis label.*

*- Regarding the scale of the IGV screen shots included in Figures 3d and 4g in the submitted manuscript and Figures 3d and 4b in the revised manuscript, the number of reads in each position was normalized against the total number of reads (reads per million mapped reads) in each sample. This is now described in the Methods section (page 18, lines 18–21) and the Figure 3d and 4b legends in the revised manuscript.*

4. In Fig. 4g, Although the manuscript is clearly written for the most part, the figures in my reviewer's copy are fuzzy and a higher resolution will be required for publication.

*Response: High-resolution figures were uploaded. In the merged file, the figures were compressed by the website. To retrieve the high-resolution images, please click the link on the upper right of the figure.*

Additional comments and suggestions are below.

The methods section is very light on detail which makes it hard to know what has been done and how robust and reproducible it is. A lot more detail would be required both for review and for publication (more detailed comments and suggestions are below).

Because I think an entire refocus of the text would benefit the manuscript, I have limited specific comments to the figures and methods section where I hope the authors will find them useful.

Specific comments:

1. Fig. 1f and g are unclear in my reviewer copy so will need better resolution for publication. The fold change scale doesn't come out at all in f, and in g it is difficult to see the differences. A different colour scale might be better.

*Response: Thank you for pointing this out. We now use a three-color-scale.*

*We also added the fold-change scales to Figure 1f and g.*

2. Fig. 3c If the text is right, the legend for lncRNA and mRNA is the wrong way round.

*Response: Thank you for this point. We corrected this mistake in the revised manuscript.*

3. Fig. 3d There's a half-seen black line above the gene id. Is this the gene model? The gene model(s) should be shown. Are the 3 panels intron-exon boundaries of one gene, windows of bases used for calling in one gene, or three different genes? See also my comments in the methods about RNAseq normalization, the right-hand scale implies that there is no cut-off for calling a read (scale 0-1 RPM with the middle panel well below 0.5) or is zero set during normalization? If so, why isn't the scale the same for all tracks as that would be a clearer illustration.

*Response: Yes. The black line above the gene id is the gene model for that gene. The other two panels present the predicted genic regions corresponding to states 1–4 based on the combinatorial pattern of epigenetic marks, which were not annotated as genes in the IWGSC RefSeq genome assembly (version 1.0). We added more details to the top of the panel and to the Figure 3d legend.*

*Regarding the scale, we used the number of reads in each position normalized against the total number of reads (reads per million mapped reads) for data normalization. The number of H3K4me1, H3K4me3, H3K36me3, and H3K9ac in these regions was relatively high. Consequently, the scale was larger than that used for the other tracks to present full peaks in the screenshot. We describe the scale in the Figure 3d legend.*

4. Fig. 3a, b, e, f, g, h and i. I found the best way to visualize these was to flip the page left and so these panels would be clearer if they were flipped.

*Response: Thank you for the suggestion. We have flipped the page and reorganized these figures, which are now clearer.*

Methods

5. Plant materials and ChIP /RNA Seq.

Were samples pooled for extraction and or analysis? If so, how many? When you say samples were analysed in duplicate, are these biological or technical replicates?

*Response: Thank you for the comment. We now describe the samples in more detail (page 16, second paragraph). For each ChIP-seq assay, 30 seedlings were collected and pooled together. To prepare biological replicates, we completed all processes separately (including sample preparation, ChIP, construction of libraries, and sequencing).*

6. Sequencing data processing.

- Please could you add more detail to the alignment method for all the sequencing? In particular, how did you ensure homeolog specificity as this is key in many parts of your study.

*Response: Thank you for pointing this out. We revised the Methods section by adding a detailed description regarding the parameters used for the alignment, mapping, and filtering (page 17, second paragraph for ChIP-seq data processing; for the BS-seq data processing, please refer to the response to the next comment). To ensure homologue specificity and accuracy for subsequent analyses, the mapping results were further stringently filtered based on the following: 1) remove reads with mapping quality (Q) < 20 [Q = −10 log10(p), where p is an estimate of the probability that the alignment does not correspond to the read’s true point of origin]. Additionally, Q > 20 ensures the reads are mostly uniquely mapped; 2) remove all supplementary alignments with a SAM flag = 2,048; 3) keep only properly mapped read pairs; and 4) remove duplicated reads mapped to exactly the same position because they are artefacts caused by PCR during library construction.*

- For the RNASeq alignment what reference transcriptome sequence was used and for the DNAse and ChIP-seq did you identify expressed genes from your own dataset (so expression in the same samples) or from the reference sequence gene models or transcriptome?

*Response: We have clarified the Methods section by indicating the source of the reference transcriptome sequence and gene models was the annotation files of the IWGSC RefSeq genome assembly (version 1.0), which were also used by Ramirez-Gonzalez et al. (2018). Please refer to page 18, lines 4-8. Regarding the definition of expressed genes, please refer to the response to the next comment.*

- What settings did you use to consider a gene expressed?

*Response: In the submitted manuscript, we used all of the high-confidence gene models in the IWGSC RefSeq genome assembly (version 1.0) for analyses, regardless of whether the gene was expressed. In the revised manuscript, we define a triad as expressed when the sum of the FPKM of homologues from three sub-genomes > 1. This definition was used for the analyses in Figure 2 (page 19, lines 16-18 and the Fig. 2c legend).*

How were RNASeq and DNAse data normalized?

*Response: To compare expression levels across samples and genes, the RNA-seq read density of each gene was normalized based on gene length and sequencing depth (i.e., fragments per kilobase of exon model per million mapped reads). For the ChIP-seq data normalization and visualization, the number of reads in each position normalized against the total number of reads (reads per million mapped reads) was used for data normalization. All relevant descriptions were added to the Methods section (page 18, lines 11-21) and the legends of Figures 3d and 4b.*

7. Bisulfite sequencing and analysis

- Were these the same samples as ChIPseq or DNase-seq?

*Response: The samples were the same as those used for the ChIP-seq and DNase-seq analyses. We clarified the description of the BS-seq experiment on page 18, lines 24-25.*

Were these sequences do-novo aligned across the whole reference genome or within gene feature models from the reference sequence how were they tiled?

*Response: These sequences were first aligned across the whole reference genome. We added the alignment parameters, including the reference genome information for the BS-seq experiment, to page 18, the 2nd paragraph. For the comparison of the methylation levels across 15 chromatin states presented in Figure 3g and Supplementary Figure 2, the average methylation ratio of each chromatin state coordinate was calculated and plotted. To plot the methylation profiles surrounding genes, we used the coordinates of the high-confidence gene models from the IWGSC RefSeq genome assembly (version 1.0). Regions from 2 kb upstream and 2 kb downstream of the gene body regions were divided into 60 tiles (20 upstream tiles, 20 downstream tiles, and 20 gene body tiles). The average methylation ratio in each tile was recorded and plotted (Supplementary Fig. 1). We clarified the Supplementary Figure 1 legend accordingly.*

- Were duplicates and ambiguous alignments removed?

*Response: We used Bismark for the BS-seq read mapping. This program reports only the best unique alignments. All non-unique alignments were removed (doi: 10.1093/bioinformatics/btr167). The quality control and read filtering are now described in the Methods section (page 18, the 2nd paragraph).*

- When you say methylation ratio was called at bases with more than 3 reads, do you mean 3 of the same reads for a cytosine (un/methylated) or do you mean any ratio?

*Response: Bases covered by fewer than three reads were considered lowconfidence positions, which were not considered for subsequent analyses.*

*The methylation ratio of a cytosine was defined as the number of mCs divided by the number of reads covering the position. This point is now described in the Methods section (page 19, the 2nd paragraph).*

8. Definition of triad genes

Were all these genes expressed in your samples? What was the cut-off for homeologous gene expression across sub-genomes?

*Response: In the submitted manuscript, we used all of the high-confidence gene models in the IWGSC RefSeq genome assembly (version 1.0) for analyses, regardless of whether the gene was expressed. In the revised manuscript, we define a triad as expressed when the sum of the FPKM of homologues from three sub-genome > 1. This definition was used for the analyses in Figure 2 (page 19, the 3rd paragraph and the Fig. 2c legend). The comparison of the homologous gene expression across sub-genomes is now described (page 20, the last paragraph).*

9. Detection of CpG Islands

How was genome GC content masked and what is the sensitivity analysis for defining the probability in the wheat genome? I.e. is 0.99 a default for much smaller and less repetitive genomes, or did you conduct a sensitivity analysis with your dataset?

*Response: We used makeCGI to predict CpG islands by fitting an HMM considering the dependence between CpG density and GC content. Thus, there was no need to mask the GC content. To determine the posterior probability cutoff, we analysed the sensitivity (Supplementary Fig. 5, page 19, the last paragraph). The receiver operating characteristic (ROC)-like plot presented the TSSs covered by CGIs (used as a measure of sensitivity) versus the total length of different CGIs (used as a measure of specificity) defined with posterior probability cutoffs ranging from 0.5 to 0.9995. We chose 0.99 as a cutoff value because it is associated with the inflection point of the ROC curve. Using 0.99 as a cutoff, we determined that 43.4% of the TSSs were covered by a CGI, which is comparable to the ratios in humans and mice (50%–56%). A subsequent comparison of the proportion of TSSs covered by random regions with the same number and length distribution of given CGIs revealed that CGIs are apparently enriched in wheat TSSs (Supplementary Fig. 5).*

10. Identification of newly formed genes

What were the criteria for a 'region' and similarity used in the MUMmer analysis? Again, what is meant by a gene here? - gene model in the reference, reference transcriptome, your own mRNA calls? How did you define low sequence identity?

*Response:*

*- We have added a detailed definition of newly formed genes (page 20, the 2nd paragraph). First, MUMmer was used to compare the genome sequences of hexaploid wheat Triticum aestivum (AABBDD sub-genome, IWGSC version 1.0) and the progenitors, including Triticum turgidum (AABB sub-genome, WEWSeq version 1.0), Triticum urartu (AA subgenome, Tu2.0), and Aegilops tauschii (DD sub-genome, ASM34733 version 2). Sequences with an identity > 90% were defined as “similar regions”. Next, to increase the stringency of the definition of old and young genes, OrthoFinder was used to detect orthologous genes between hexaploid wheat and the progenitors. Orthologous genes within similar regions (sequence identity > 90%) in hexaploid wheat and the progenitors were defined as “old” genes (100,166 genes), whereas hexaploid wheat genes outside the similar regions and without orthologues in wild progenitors were defined as “young” genes (10,624 genes).*

*- All gene models were downloaded from published annotated reference genome sequences. Details regarding the genomic resources are listed in the Methods section (page 20, lines 17-24).*

11. Detection of transcription factor binding motifs

Which gene models are used and how do you define the promoter and enhancer-like regions? What was the total number of scanned regions and how is enrichment defined? What is the size (bp) and distribution of the scanned regions in the genome? Is this where the 1.5% comes from? I'd lie to know how that's calculated and how it compares to what are defined here as promoter and genic regions. Currently it's hard to know whether the percentage is informative what you mean by 'large' regions of regulation.

*Response: We now describe the detection of transcription factor binding motifs in greater detail (page 11, lines 32 and page 12, lines 1–3 in the Results section; page 22, the 3rd paragraph in the Methods section; Figure 5e legend). Active promoter and enhancer-like regions used for motif analyses were from state 5, which was highly conserved regarding sequences and epigenetic activities across sub-genomes. Specifically, regions in state 5 within 3 kb upstream of TSSs were defined as promoters, whereas other intergenic regions in state 5 were defined as enhancer-like regions. A total of 46,834 proximal (promoter) and 23,563 distal (enhancer-like) regions were detected and used to identify enriched motifs. The enrichment was defined as the number of occurrences for a given motif in the Jaspar database normalized against the number of promoter or enhancer-like regions. Chromatin states 5–7 represented 1.5% of the genome (i.e., the last column in Figure 3a represents the genome coverage of a given state). We now describe how 1.5% was calculated on page 9, lines 18–21. We agree that the use of “large” regions was improper. This was changed to “a subset” (page 3, line 14 and page 13, line 9).*

**Reviewer 2**

The bread wheat epigenomic map reveals distinct chromatin architectural and evolutionary features of functional genetic elements" by Li et al. submitted to Genome Biology.

In this work, the authors performed transcriptomic analysis and whole genome profiling of seven chromatin modifications in hexaploid bread wheat genome. Based on these data, the authors developer open access genome-wide maps of individual modifications. They confirmed generally euchromatic or heterochromatic nature of individual modifications and identified their association with specific chromosome regions. This created very rich dataset for analysis of transcriptional activity of individual genes with respect to chromatin modifications, effect of individual wheat subgenomes and evolutionary conservation. Finally, they identified regions containing putative promoter and enhancer elements and in one specific case also validated their prediction using orthologous system of Nicotiana benthamiana.

In general, this is very timely and useful study on economically important species containing very large genome and therefore technically difficult. The writing is clear with no mistakes. The bioinformatics is high level. The study, as such, is mostly descriptive with only small (final) part, which is experimental.

Here comes my main concern.

1. The authors tested six predicted enhancer regions from one genomic locus. Out of these six, 50% stimulated expression from the neighboring promoter, while the rest did not. Based on this it is hard to guess whether this is typical situation or other regions will be more successful. I would very much appreciate testing few more predicted enhancers to get better estimate on the reliability of this perdition. Can the interactions also be influenced by the fact that the monocot regions are tested in a dicot system.

*Response: Thank you for the comments and suggestions.*

*- In this study, 50% was a stringent estimation because only regions that exhibited a robust enhancer activity in all spots and in all replicates were counted. Each experiment was replicated three times, and each replicate included three leaf spots. Regions exhibiting enhancer activity in only some of the replicates were excluded, but may also have a weak or dynamic enhancer activity as discussed (page 15, the last paragraph in the Discussion section). The active cis-regulatory regions predicted in this study may require additional temporal or spatial trans-acting factors for the fulfilment of the enhancer function. Additionally, considering the large bread wheat genome and the complex interaction between the three subgenomes, there may be abundant inter- and long distance intrachromosomal interactions that are coordinated to regulate transcriptional activity. The cis-elements predicted in the present study represent good candidates for further studies of the transcriptional regulation by large-scale chromosomal interactions.*

*- We experimentally validated an additional 20 regions in state 5 and six regions in states 1 and 2. We observed that 50% of the regions exhibited robust activity in all three replicates. We also calculated the correlation between the signal densities from the reporter assay with the read densities of each epigenetic mark in the tested regions (Fig. 6d and e). The DHS and H3K9ac densities were the most highly correlated with enhancer activity (r = 0.5 and 0.3, respectively), indicating these marks are good for predicting enhancer activity. Our findings further support the use of chromatin signatures to identify genomic regions with enhancer functions.*

*- To analyse a monocot system, we transiently transformed wheat leaf protoplasts with the six experimentally validated regions. The subsequent observations were similar to those of a transient transfection of Nicotiana benthamiana leaves (the following figure vs. Fig. 6b). We repeated this experiment three times and consistently obtained positive results for the first three predicted enhancers. We did not use a protoplast system for further validations because quantifying the activity requires not only the number of fluorescent protoplasts, but also the total number of protoplasts, thus making the quantification and comparison impractical. The results of the luciferase reporter assay for the N. benthamiana leaves was similar to the* *results for the wheat protoplasts. The former is an in vivo system whose activity is relatively easy to quantify. Consequently, we used the luciferase reporter assay for all additional experimental validations (Fig. 6, Supplementary Fig. 4, page 12, the last paragraph).*

2. In the first section of the results, the authors claim that H3K4me1 has not been functionally characterized in plants. I would like to draw their attention to the study by Zhang et al, Genome Biol, 2009, where this has been done in Arabidopsis (at least to the level presented here).

*Response: Thank you for pointing this out. We have added a description of the findings of the Zhang et al. study to the revised manuscript (page 6, lines 7–9).*

Minor comments:

1. twice there is written „subgenomes increased the flexibility and adaptability of bread wheat". Please specify to what.

*Response: Thank you for this comment. We have added a more detailed description about the flexibility and adaptability (page 4, lines 31–32; page 5, lines 1–2; page 7, lines 16–18).*

2. the first page of discussion, line 38 - error in reference format

*Response: Thank you for pointing this out. The mistake has been corrected in the revised manuscript (page 14, line 3).*

3. the first page of discussion, line 40 - space missing

*Response: Thank you for pointing this out. This sentence has been re-written (page 14, lines 4–7).*

**Second round of review**

**Comments to author and authors’ point-by-point responses to the reviewers’ comments:**

**Reviewer 1**

Accept

**Reviewer 3 (in replacement of Reviewer 2)**

Li et al. presented a comprehensive study of chromatin states, epigenomic marks and transcriptomic data in the bread wheat genome. Epigenomic features were characterized throughout the three subgenomes of the allohexaploid wheat, and genes with similar epigenetic modifications were further characterized in various ways. They also annotated long-distance regulatory elements based on the combination of several chromatin signatures, which would be of interest to research scientists in wheat functional genomics field. In general, I would agree with the comments from the two previous reviewers that all helped to improve the manuscript. I think the current version of the manuscript and the authors' response have addressed the reviewers' comments professionally and properly. Even though most of the results in this manuscript are descriptive to some extent, I think it is still a timely study regarding wheat epigenomic map. A focused epigenetic study of a specific aspect of wheat biology could be built on this overview pattern of chromatin profiling in wheat. Several specific comments as following: 1.Given the epigenomic features sometimes differ/switch states across different tissues and development stages (as in fig 1e), it is better mention about the specific plant materials used for sequencing either in the abstract or the first part of the Results.

*Response: Thank you for the suggestion. We added the description of plant materials used for sequencing to BOTH Abstract and the first part of the Results (page 3, line 10; page 5, line 26; and page 6, line 1).*

2.The authors predicted some cis-elements either proximal or distal ones, but didn't mention about the expression status of the nearby genes. Is that possible to predict the regulated genes of these cis-elements? A catalog of predicted cis-regulatory elements and the potential regulated gene would be much helpful.

*Response: Thank you for the comments and suggestions. We included these information in the revised Supplementary Table 4a and 4b. Specifically, 1) we assigned the putative target genes of these cis-elements based on proximity; 2) we listed the expression status of each target gene.*

3.It is also interesting that 8,987 potentially missed genes in the current annotation of CS. Are these genes in the low confidence set? Are the translation frames in good shape?

*Response: Thank you for the comments and suggestions. We observed that out of the 8,987 potentially missed genes in the high confidence genes of CS, 63% were annotated as low confidence genes. Among the rest transcripts, 94% have low coding potential, i.e. non-coding transcript. We revised the manuscript accordingly (Results: page 9, lines 4-9; Methods: page 18, lines 12-13 and page 22, the 1st paragraph), and added all these information to Supplementary Table 3.*
